# Novel anxiolytic-antidepressant combinations TF-1 and TF-2 and their intervention mechanisms in a mouse model of comorbid anxiety and depression (CAD)

**DOI:** 10.3389/fphar.2026.1866088

**Published:** 2026-07-06

**Authors:** Min Wang, Yuxiang Wang, Zheng Li, Rongzhu Li, Liu Wang, Lin Sheng, Peng Shi, Ying Zuo, Yumeng Wei, Ling Zhao

**Affiliations:** 1 Luzhou Key Laboratory of Traditional Chinese Medicine for Chronic Diseases Jointly Built by Sichuan and Chongqing, School of Pharmacy, Southwest Medical University, Luzhou, China; 2 Luzhou Key Laboratory of Traditional Chinese Medicine for Chronic Diseases Jointly Built by Sichuan and Chongqing, The Affiliated Traditional Chinese Medicine Hospital, Southwest Medical University, Luzhou, Sichuan, China; 3 Central Nervous System Product Research and Development Key Laboratory of Sichuan Province, School of Pharmacy, Southwest Medical University, Luzhou, Sichuan, China; 4 Department of Comprehensive Medicine, The Affiliated Traditional Chinese Medicine Hospital, Southwest Medical University, Luzhou, Sichuan, China; 5 Department of Psychiatry, Fundamental and Clinical Research on Mental Disorders Key Laboratory of Luzhou, The Affiliated Hospital, Southwest Medical University, Luzhou, Sichuan, China

**Keywords:** anxiety, compositions, depression, fluoxetine, tandospirone

## Abstract

**Background and Objective:**

To address the high mortality, slow onset, and suboptimal efficacy of current treatments for comorbid anxiety and depression (CAD), this study developed and systematically evaluated a novel combination formulation.

**Methods:**

The anxiolytic tandospirone (TDS) was combined with fluoxetine (FLU), escitalopram, or milnacipran for cell-based screening. The optimal combination was selected for pharmacokinetic studies in rats, followed by a 49-day pharmacodynamic evaluation and mechanistic investigation in a CAD mouse model.

**Results:**

Two optimal TDS-FLU combinations, TF-1 (TDS:FLU = 1.2:1,w/w) and TF-2 (TDS:FLU = 2.4:1,w/w), were obtained, both exhibiting good neuroprotective effects. Pharmacokinetic results showed that TF-1 and TF-2 significantly increased drug exposure and prolonged half-life compared with monotherapies of TDS and FLU. Pharmacodynamically, TF-1 and TF-2 exhibited superior anxiolytic-antidepressant efficacy compared with monotherapies after 28-day and 49-day interventions. At day 28, both formulations significantly increased open-field central-zone activity and sucrose preference, and reduced immobility time (all P < 0.05). Their therapeutic benefits were further augmented at day 49, with sustained and improved behavioral improvements upon long-term treatment. Mechanistic studies showed that both combinations elevated plasma and hippocampal 5-HT and BDNF levels, upregulated hippocampal 5-HT1AR and TrKB expression, and enhanced neuronal plasticity.

**Conclusion:**

TF-1 and TF-2 were superior to single agents in pharmacokinetics and long-term CAD efficacy, acting via 5-HT modulation and BDNF-TrKB activation, supporting their development as CAD combination therapies.

## 1 Introduction

Depression is a heterogeneous psychiatric disorder characterized by disturbances in appetite, sleep, mood, thinking, interest, and somatic functions (Monroe and Harkness, 2022; Thapar et al., 2022; Chen et al., 2026). According to data released by the World Health Organization, the number of individuals with depression worldwide has exceeded 350 million (Rozov et al., 2024),and depression is projected to become the leading cause of global disease burden by 2030 (Crawford et al., 2024). In clinical practice, depression frequently coexists with anxiety disorders. Accumulating evidence has demonstrated that up to 60% of patients with major depressive disorder experience lifelong comorbid anxiety disorders, which are predominantly manifested as depressive symptoms accompanied by anxiety states of varying severities (Dong C. et al., 2022; Meng et al., 2025). This comorbid condition has been formally defined as comorbid anxiety and depression (CAD) in the latest revision of the International Classification of Diseases (ICD-11), referring to a clinical condition with the simultaneous occurrence of anxiety and depressive disorders (ICD-11 for Mortality, 2026). CAD defined by ICD-11 is an independent mood-disorder entity, whereas DSM-5 classifies its corresponding clinical phenotype using the “with anxious distress” specifier rather than establishing a separate diagnosis (ICD-10 Version, 2019).

In terms of pathological mechanisms, the onset and progression of CAD are closely associated with serotonin (5-HT) system dysfunction, endocrine disorders, genetic susceptibility, environmental factors, and neurotic personality traits (Goodwin, 2021). Neurobiological studies have indicated that the core pathogenesis of CAD stems from the disrupted balance of neural impulses in the brain limbic system. Compared with patients suffering from a single disorder, CAD patients present more complicated clinical manifestations, severe cognitive impairment, and frequent unexplained somatic symptoms. Additionally, they are featured by later disease onset, higher suicide risk, and more severe impairment in social and occupational functions (Groen et al., 2020; Bobo et al., 2022; Kazemi et al., 2025). In terms of clinical treatment, CAD patients generally exhibit poor responses to pharmacotherapy, including delayed onset of therapeutic action, increased incidence of treatment resistance and high recurrence risk, thereby posing severe challenges for clinical diagnosis and management.

Nevertheless, first-line antidepressants widely applied in current clinical practice exert limited therapeutic effects on CAD. Such pharmacological agents are accompanied by slow therapeutic onset and may elevate potential health risks in patients (Peleg et al., 2022; Tran et al., 2025; Frey et al., 2026). Therefore, the development of novel and optimal therapeutic drugs for CAD is urgently required in clinical settings. At present, combined medication serves as a conventional clinical strategy to improve the treatment efficacy of CAD (Chen et al., 2024; Chrysafi et al., 2024; Duo and Rao, 2024). Rational drug combinations are expected to enhance therapeutic effects via synergistic mechanisms, shorten the onset time of action, and reduce adverse reactions (Takeshima et al., 2022). However, inappropriate combined medication may also lead to polypharmacy abuse, increased drug tolerance, treatment resistance, and declined patient compliance (Huang et al., 2020). Accordingly, exploring rationally proportioned combination drugs with complementary mechanisms and controllable safety profiles is a critical direction to optimize CAD treatment.

Tandospirone citrate (TDS) is a first-line anxiolytic agent widely used in clinical practice for the treatment of various chronic anxiety disorders (Huang et al., 2017; Fu et al., 2023; Li R. et al., 2023). As a selective 5-HT_1_A receptor agonist, TDS possesses anxiolytic efficacy comparable to diazepam, a classic benzodiazepine drug. Notably, TIS exhibits distinct superiorities including no addiction, drug tolerance or withdrawal reactions, and exerts pharmacological effects by selectively activating and inducing the desensitization of central 5-HT_1_A receptors (Zhan et al., 2022). Given its unique mechanism of action, TDS is commonly utilized as an adjuvant agent for antidepressants. Existing studies have verified that the combined administration of TDS and antidepressants can markedly strengthen therapeutic efficacy, accelerate drug action, and alleviate adverse reactions (Lin et al., 2018; Chen et al., 2025b). These findings suggest that compound preparations composed of TDS and antidepressants may act as a promising therapeutic strategy for CAD.

Hence, the current study focused on optimizing the pharmacokinetic properties and enhancing the therapeutic efficacy against CAD. Firstly, TDS was combined with three clinically commonly used antidepressants, namely, fluoxetine (FLU), escitalopram (ESC) and milnacipran (MIL), followed by preliminary screening through *in vitro* antidepressant activity evaluation. Subsequently, two novel TDS-FLU compound formulations, TF-1 (TDS: FLU = 1.2:1, w/w) and TF-2 (TDS: FLU = 2.4:1, w/w), were selected for subsequent detection of pharmacokinetic characteristics, verification of *in vitro* and *in vivo* pharmacodynamics, and exploration of the underlying mechanisms. This study aims to clarify the pharmacokinetic advantages and synergistic anti-CAD potential of TF-1 and TF-2, and to provide pivotal experimental evidence for the development of novel compound preparations with optimized pharmacokinetic behaviors and potent anti-CAD activity.

## 2 Materials and methods

### 2.1 Reagents

Tandospirone citrate (TDS, Kedead Pharmaceutical Co., Ltd., Sichuan, China); Fluoxetine (FLU), Escitalopram (ESC) and Mirtazapine (MIL) (Maclean Biochemical Technology Co., Ltd., Shanghai, China); Glutamate (Glu, Aladdin Biochemical Technology Co., Ltd., Shanghai, China); MTT (Solarbio Science & Technology Co., Ltd., Beijing, China); Lactate dehydrogenase (LDH) colorimetric assay kit (Nanjing Jiancheng Bioengineering Institute); Absolute ethanol (analytical grade, Xilong Science Co., Ltd., Sichuan, China); Fetal bovine serum (Thermo Fisher Scientific, Beijing, China).

### 2.2 Cells and experimental animals

The highly differentiated PC12 cells used in this study, which were pre-differentiated by nerve growth factor (NGF), were purchased from the Cell Bank of the Chinese Academy of Sciences. Four-week-old male C57BL/6 mice were obtained from Tengxin Experimental Animal Co., Ltd., Chongqing, China (License No.: SCXK (Jing) 2019–0,010). All mice were raised in a specific pathogen-free (SPF) environment at a temperature of (25 ± 2) °C and humidity of 40%–60%, and housed in single cages during modeling and drug administration. All animal experiments were approved by the Animal Ethics Committee of Southwest Medical University (Approval No.: 20,220,825–002). Mice were anesthetized via intraperitoneal injection of sodium pentobarbital and euthanized by cervical dislocation.

### 2.3 Screening of *in vitro* neuroprotective effects of TDS combinations

#### 2.3.1 Establishment of glutamate-induced cell injury model

Referring to the reported method (Virtanen et al., 2020), PC12 cells in the logarithmic growth phase were seeded into 96-well plates. After cell adherence, different concentrations of glutamate (Glu) were added for 24 h of intervention. MTT solution (5 mg/mL) was added to each well and incubated for 4 h. The supernatant was discarded, and DMSO was added to dissolve formazan crystals. The absorbance (A value) was measured at 490 nm (or 570 nm), and cell viability was calculated. The Glu concentration (10 mM) that reduced cell viability to 50%–60% was selected to establish the injury model.

#### 2.3.2 Screening of safe concentrations of single agents

The MTT assay was used to detect the viability of PC12 cells treated with gradient concentrations of TDS, FLU, ESC and MIL for 24 h. The concentration-viability curves were plotted to determine the maximum safe concentration of each monotherapy, which was set as the upper concentration limit for combined administration.

#### 2.3.3 Design of combined administration regimens

Based on the IC_50​_ ratio of each single drug, the fixed-ratio method was adopted to design compound ratios of TDS combined with FLU (TF), ESC (TE) and MIL (TM), including core ratios close to the IC_50​_ ratio (TF 2.4:1, TE 1:3, TM 1:4), gradient control ratio (TF 1.2:1) and equal mass ratio control (1:1). The experimental groups were set as follows: normal control group (complete medium), Glu model group (10 mM Glu), monotherapy control groups (single drug +10 mM Glu), and combined administration groups (compounds at above ratios +10 mM Glu). The concentration of each single component in combination groups did not exceed its maximum safe concentration.

#### 2.3.4 Evaluation of synergistic neuroprotection (combination index, CI)

After 24 h of intervention according to the above grouping, cell viability was detected by MTT assay. The Chou-Talalay median-effect principle was applied to calculate the combination index (CI). A CI value <0.8 was defined as synergistic effect, and a lower CI indicated stronger synergism. The ratios with significant synergistic effects were screened for subsequent studies.

#### 2.3.5 Determination of LDH release rate

Cells in the logarithmic growth phase were seeded into 96-well plates and treated with the optimized ratios for 24 h. The cell supernatant was collected, and the absorbance was detected at 440 nm in accordance with the instructions of the LDH colorimetric assay kit. LDH activity was calculated by the corresponding formula to evaluate cell membrane integrity. LDH release was detected using an LDH cytotoxicity assay kit, and the results were normalized to total cellular protein content.

### 2.4 *In vivo* pharmacokinetic study of TF combinations

#### 2.4.1 Animals and administration

A total of 25 healthy male SD rats were used. After adaptive feeding, rats were randomly divided into five groups (n = 5). All animals were fasted for 12 h with free access to water before the experiment. Intragastric administration was performed as follows: low-dose TDS monotherapy group (48 mg/kg), high-dose TDS monotherapy group (96 mg/kg), FLU monotherapy group (40 mg/kg), TF-1 combination group (48 mg/kg TDS +40 mg/kg FLU), and TF-2 combination group (96 mg/kg TDS +40 mg/kg FLU).

#### 2.4.2 Plasma sample collection

Approximately 0.3 mL of blood was collected from the orbital venous plexus or caudal vein at 0.083, 0.25, 0.33, 0.5, 1, 2, 4, 8, 10, 12, 24 and 48 h after administration. Blood samples were placed in heparin-pretreated centrifuge tubes. Whole blood was centrifuged at 6,000 rpm for 3 min to separate plasma, which was stored at −20 °C or −80 °C for subsequent analysis.

#### 2.4.3 Sample analysis and data processing

After pretreatment, plasma samples were determined by HPLC to detect the concentrations of TDS and FLU. The mean plasma concentration-time curves were plotted. The non-compartmental model in DAS 2.1.1 software was used to calculate major pharmacokinetic parameters. The pharmacokinetic interactions after combined medication were evaluated by comparing parameters between monotherapy groups and TF combination groups.

### 2.5 *In vivo* anti-anxiety and anti-depression effects and preliminary mechanism of TF

#### 2.5.1 Establishment of comorbid anxiety and depression (CAD) model

Referring to the method (Moreno-Fernández et al., 2023), the CAD model was established by chronic unpredictable mild stress (CUMS) combined with single-cage rearing. After 5 days of adaptive feeding, mice with normal behavioral performance were divided into normal control group and CUMS model group, and mice in the CUMS group were raised in isolation (Song et al., 2021). Animals were randomly assigned to experimental groups using a random number table generated by SPSS 26.0. Blinding was strictly performed during behavioral testing and data analysis: experimenters conducting behavioral assessments and data analysts were kept unaware of group allocation throughout the study. Sample size was estimated via power analysis using G*Power 3.1 software (α = 0.05, 1-β = 0.8, Cohen’s d = 0.8), and eight mice per group were included to ensure statistical reliability.

One to two types of unpredictable stresses were given daily without continuous repetition of the same stimulus (Zhou et al., 2020; Xie et al., 2022), including hot/cold water swimming (40 °C/4 °C, 5 min), noise stimulation (1 h), shaking treatment (15 min), tilted cage (24 h), odor stimulation (4 h), tail suspension (6 min), 50 mL centrifuge tube restraint (4 h), wet bedding (24 h), electric shock (1 min), food/water deprivation (24 h), continuous illumination (48 h) and continuous darkness (48 h). All stress interventions were terminated 12 h before behavioral tests, with regular feeding maintained, the detailed stress schedule is shown in Table 1.

**TABLE 1:** Detailed schedule of chronic unpredictable mild stress (CUMS) interventions for 50-day model establishment.

Modeling day	Intervention method	Modeling day	Intervention method
1	Hot water swimming (40 °C, 5 min)	26	Tail suspension (6 min)
2	Wet bedding (24 h)	27	Noise stimulation (1 h)
3	Electric shock (1 min)	28	50 mL centrifuge tube restraint (4 h)
4	Shaking treatment (15 min)	29	Wet bedding (24 h)
5	Tail suspension (6 min)	30	Electric shock (1 min)
6	Tilted cage (24 h)	31	Shaking treatment (15 min)
7	50 mL centrifuge tube restraint (4 h)	32	Odor stimulation (4 h)+continuous darkness (48 h)
8	Cold water swimming (4 °C, 5 min)	33	Hot water swimming (40 °C, 5 min)
9	Odor stimulation (4 h)+continuous darkness (48 h)	34	Tail suspension (6 min)
10	Shaking treatment (15 min)	35	50 mL centrifuge tube restraint (4 h)
11	Wet bedding (24 h)	36	Wet bedding (24 h)
12	Electric shock (1 min)	37	Electric shock (1 min)
13	Tail suspension (6 min)	38	Shaking treatment (15 min)
14	Cold water swimming (4 °C, 5 min)	39	Odor stimulation (4 h)+continuous darkness (48 h)
15	Odor stimulation (4 h)+continuous illumination (48 h)	40	50 mL centrifuge tube restraint (4 h)
16	Water deprivation (24 h)	41	Tail suspension (6 min)
17	Shaking treatment (15 min)	42	Cold water swimming (4 °C, 5 min)
18	Wet bedding (24 h)	43	Odor stimulation (4 h)+continuous illumination (48 h)
19	Electric shock (1 min)	44	Shaking treatment (15 min)
20	Tail suspension (6 min)	45	Noise stimulation (1 h)
21	Odor stimulation (4 h)+continuous illumination (48 h)	46	Tilted cage (24 h)
22	Hot water swimming (40 °C, 5 min)	47	Hot water swimming (40 °C, 5 min)
23	Shaking treatment (15 min)	48	Cold water swimming (4 °C, 5 min)
24	Wet bedding (24 h)	49	Shaking treatment (15 min)
25	Electric shock (1 min)	50	Wet bedding (24 h)

#### 2.5.2 Grouping and drug administration

After behavioral evaluation, mice with depression- and anxiety-like phenotypes were randomly assigned into six groups: CAD model group, FLU-7.5 group (7.5 mg/kg/d), TDS-9 group (9 mg/kg/d), TDS-18 group (18 mg/kg/d), TF-1 group (7.5 mg/kg FLU +9 mg/kg TDS) and TF-2 group (7.5 mg/kg FLU +18 mg/kg TDS), with 10 mice in each group. Mice in the normal control and CAD model groups were given sterile water. After 28 days (4 weeks) of intragastric administration, the first behavioral tests were conducted on Day 29. Partial mice were sacrificed for tissue collection, and the remaining mice received continuous administration for another 21 days until Day 49 for the second behavioral evaluation. After the final test, blood was collected from the inner canthus, centrifuged at 3,500 rpm for 15 min to separate plasma and stored at −80 °C. Brain tissues were harvested after cervical dislocation, and hippocampal tissues were isolated and preserved at −80 °C. 2.5.3 Behavioral tests.

#### 2.5.3 Behavioral tests

1. Open field test (OFT): After 30 min of environmental adaptation, mice were placed in an open field box (50 cm × 50 cm × 40 cm). The central region movement distance and residence time within 5 min were recorded.
2. Elevated plus maze (EPM): Mice were placed in the central area facing the open arms. The entry times and residence time in open/closed arms within 5 min were recorded. (1) Open field test (OFT): After 30 min of environmental adaptation, mice were placed in an open field box (50 cm × 50 cm × 40 cm). The central region movement distance and residence time within 5 min were recorded.
3. Sucrose preference test (SPT): After adaptive training, drinking water and 2% sucrose solution were supplied simultaneously. The fluid consumption within 12 h was recorded to calculate the sucrose preference rate (Zhang M. et al., 2022).
4. Forced swimming test (FST): Mice were placed in 25 °C water with a depth of 10 cm for 1 min of adaptation, and the immobility time within the subsequent 5 min was recorded (Wang C.-C. et al., 2021).

#### 2.5.4 Enzyme-linked immunosorbent assay (ELISA)

Hippocampal tissues were homogenized with PBS (1:9, w/v) and centrifuged. The supernatant was collected to detect the contents of 5-HT and BDNF according to the kit instructions, and the absorbance was measured at 450 nm.

#### 2.5.5 Quantitative real-time polymerase chain reaction (qPCR)

Total RNA was extracted from hippocampal tissues with Buffer RL1. After genomic DNA removal and reverse transcription, qPCR amplification was performed.

#### 2.5.6 Western blot

Hippocampal tissues were lysed with RIPA lysis buffer (1:10, w/v) at 4 ^°^C for 30 min, followed by centrifugation at 12,000 rpm for 10 min. Protein concentration was determined by quantitative assay. After electrophoresis, membrane transfer and blocking, membranes were incubated with primary antibodies (5-HT_1_AR, TrKB) and corresponding secondary antibodies. Protein bands were visualized by ECL chemiluminescence, and grayscale analysis was conducted.

#### 2.5.7 Hepatic and renal function detection

Plasma samples were collected after 49 days of administration. The levels of ALT, AST, CREA and UREA were determined by a veterinary biochemical analyzer.

#### 2.5.8 Hematoxylin-eosin (HE) staining

Hippocampus, heart, liver and kidney tissues were fixed in 10% neutral formalin, followed by dehydration, embedding and sectioning. Tissue sections were stained with HE, sealed and observed for pathological changes using a Pannoramic 250 scanner.

### 2.6 Statistical analysis

Statistical analysis was performed using GraphPad Prism 7.0. Software. First, the normality of data distribution and homogeneity of variance were assessed by Shapiro-Wilk test and Levene’s test, respectively. Student’s t-test was used for comparisons between two groups. For single-time-point multi-group comparisons, one-way analysis of variance (one-way ANOVA) was conducted, followed by Tukey’s *post hoc* multiple comparison test. Repeated-measures one-way ANOVA with Tukey’s *post hoc* test was applied for longitudinal data obtained from the same mice at day 28 and day 49. All data were expressed as mean ± standard deviation (x ˉ± s), and P < 0.05 was considered statistically significant.

## 3 Results

### 3.1 Screening of neuroprotective effects of TDS combinations

#### 3.1.1 Safe concentrations of single drugs in PC12 cells

**FIGURE 1:**
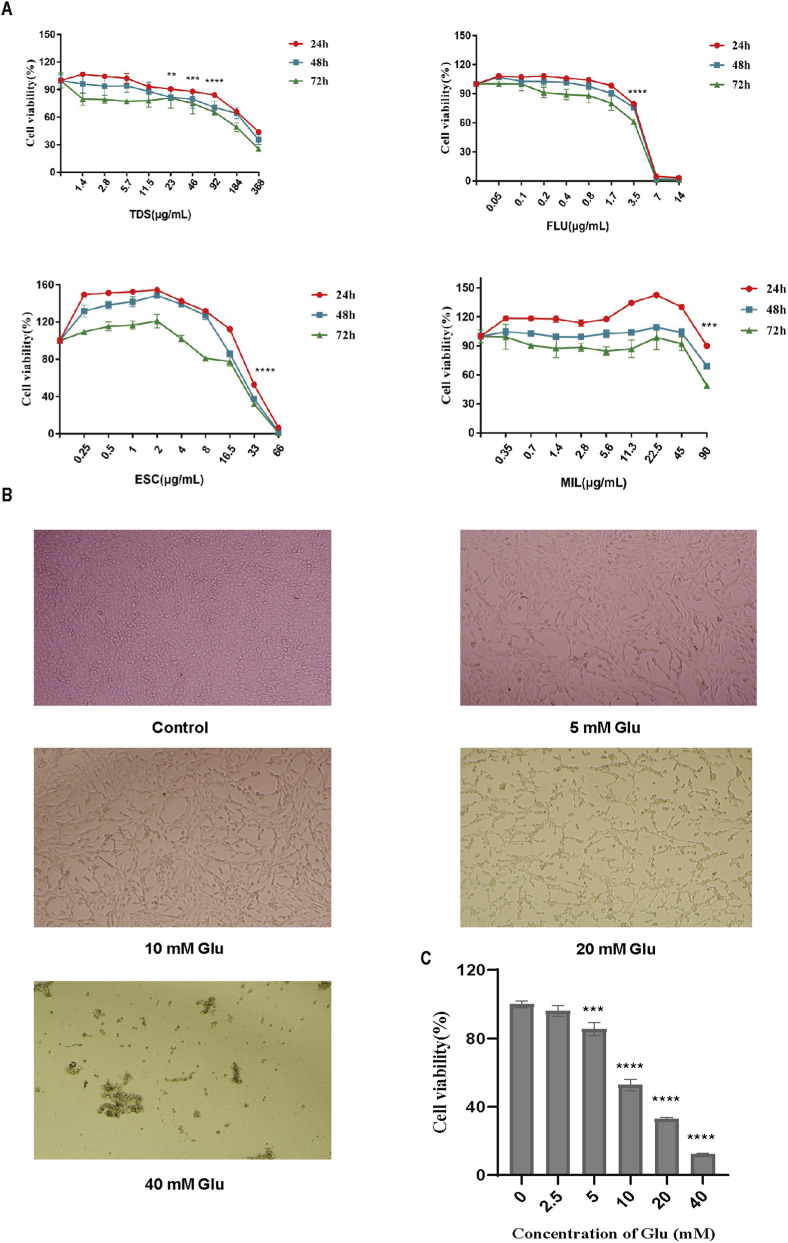
**(A)** The survival rate of TDS, FLU, ESC and MIL on PC12 cell. **P < 0.01, ***P < 0.001, ****P < 0.0001, compared with the normal control group. **(B)** Morphological changes of PC12 cell under different concentrations of Glu. **(C)** The survival rate of PC12 cell treated with different concentrations of Glu **(B)** ***P < 0.001, ****P < 0.0001 compared with the normal control group.

PC12 cells were treated with gradient concentrations of TDS, FLU, ESC and MIL for 24, 48 and 72 h, respectively. As shown in Figure 1A, the inhibitory effects of the four drugs on PC12 cell proliferation exhibited concentration- and time-dependent characteristics. Specifically, cell viability decreased continuously with the increase of drug concentration and the prolongation of treatment duration. Taking cell viability higher than 90% as the safety screening criterion, the maximum safe concentrations of each drug after 24 h intervention were determined as follows: TDS 11.5 μg/mL, FLU 1.7 μg/mL, ESC 16.5 μg/mL, and MIL 45 μg/mL. Concentrations exceeding the above ranges significantly reduced cell viability compared with the blank control group (P < 0.01, P < 0.001, P < 0.0001). Accordingly, 24 h was selected as the intervention duration for subsequent combined administration. The maximum safe concentrations of each drug at 24 h were set as the upper dosage limits to further explore the protective effects of TDS compound combinations on glutamate-induced PC12 cell injury.

#### 3.1.2 Establishment of glutamate-induced PC12 cell injury model

PC12 cells were exposed to gradient concentrations of glutamate (5, 10, 20 and 40 mM) for 24 h, and the cell morphology and viability were presented in Figures 1B,C. Microscopic observation revealed that cells in the normal control group exhibited full morphology, clear synapses and uniform distribution. With the increase in glutamate concentration, cells gradually shrank, synapses atrophied and disappeared, and intercellular spaces widened. A large number of cell debris and suspended dead cells were observed in the 40 mM group (Figure 1B). MTT results demonstrated that glutamate inhibited PC12 cell viability in a concentration-dependent manner, and the cell viability of all glutamate-treated groups was significantly lower than that of the normal control group. The cell viability was (85.42 ± 3.80) % for the 5 mM group (52.81 ± 3.29) % for the 10 mM group (32.95 ± 0.85) % for the 20 mM group, and (12.55 ± 0.18) % for the 40 mM group. The half-maximal inhibitory concentration (IC_50_​) of glutamate against PC12 cells was calculated to be 12.18 mM by nonlinear fitting. To establish a moderate injury model with cell viability ranging from 50% to 60% for evaluating the neuroprotective effects of drugs, 10 mM glutamate was selected to induce PC12 cell injury in subsequent experiments.

#### 3.1.3 Effects of TDS combinations on the proliferative activity of glutamate-injured cells

**FIGURE 2:**
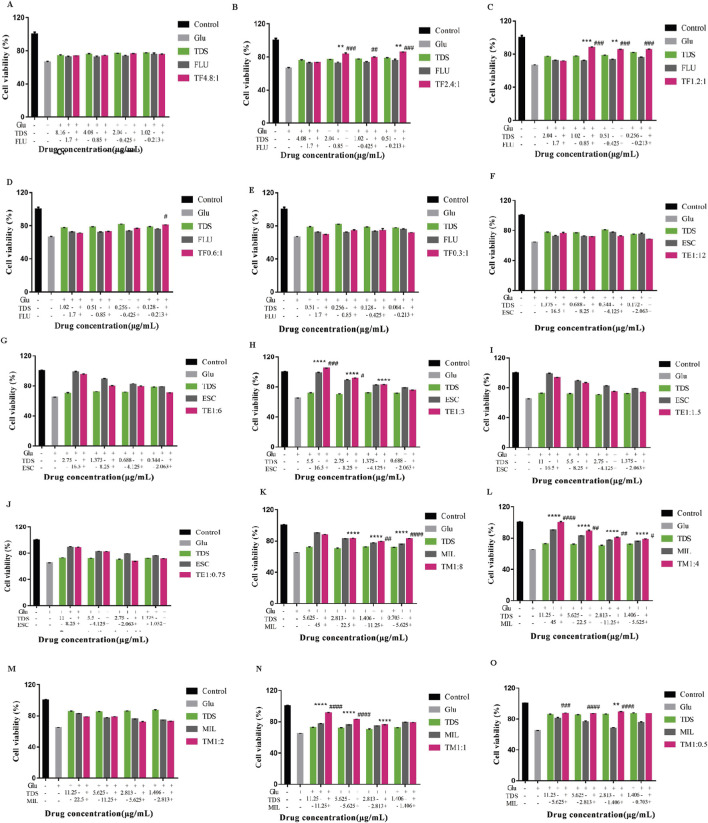
Effects of TDS compositions with different ratios on cell viability in Glu-induced injured PC12 cells **(A–O)** Cell viability of Glu-induced injured PC12 cells treated with TDS compositions. The TDS compositions were prepared at the following ratios, respectively: TF4.8:1 **(A)** TF2.4:1 **(B)** TF1.2:1 **(C)** TF0.6:1 **(D)** TF0.3:1 **(E)** TE1:1.2 **(F)** TE1:6 **(G)** TE1:3 **(H)** TE1:1.5 **(I)** TM1:8 **(J)** TM1:3.5 **(K)** TM1:4 **(L)** TM1:2 **(M)** TM1:1 **(N)** TM1:0.5 **(O)** Statistical analysis: Compared with the TDS group, *P < 0.05, **P < 0.01, ***P < 0.001, ***P < 0.0001; compared with the FLU, ESC and MIL groups, #P < 0.05, ##P < 0.01, ###P < 0.001, ####P < 0.0001.

Based on the safe concentrations of single drugs and the 10 mM glutamate injury model, injured cells were treated with TDS combinations containing different ratios (TF, TE and TM). Cell viability was detected and the combination index (CI) was calculated, as shown in Figure 2, Table 2. Compared with the glutamate model group, monotherapy with TDS, FLU, ESC or MIL exerted a weak improvement in cell viability, whereas all TDS combination groups upregulated cell activity to varying degrees. The optimal ratios were TF (2.4:1, 1.2:1), TE (1:3), and TM (1:4, 1:1). The CI values at different effect levels were calculated according to the Chou-Talalay median-effect principle, and a CI value <0.8 was defined as a synergistic effect. The CI values of the above optimal ratio groups were all less than 0.8, confirming that TDS exerted synergistic neuroprotective effects against glutamate-induced PC12 cell injury when combined with FLU, ESC and MIL at the corresponding ratios.

**TABLE 2:** Combined index (CI) of protective effect of TF with different mass ratios on PC12 injured cell.

Drug-combination	Mass ratio	Fa	CI	Combined effect
TF	4.8:1	0.75	》1.2	Antagonism
	2.4:1		0.03	Synergism
	1.2:1		0.32	Synergism
	0.6:1		》1.2	Antagonism
	0.3:1		》1.2	Antagonism
TE	1:12	0.9	1.42	Antagonism
	1:06		2.23	Antagonism
	1:03		0.7	Synergism
	01:01.5		2.37	Antagonism
	01:00.8		2.88	Antagonism
TM	1:08	0.85	0.98	Additive
	1:04		0.36	Synergism
	1:02		1.71	Antagonism
	1:01		0.18	Synergism
	01:00.5		1.38	Antagonism

Combining the results of cell viability and CI analysis, five optimal compound ratios were screened for subsequent experiments: TF (TDS: FLU = 2.4:1, 1.2:1), TE (TDS: ESC = 1:3), and TM (TDS:MIL = 1:4, 1:1). Further verification with gradient concentrations showed that these five combinations markedly increased the viability of injured cells with CI values below 0.8, indicating significant synergistic protective effects. Therefore, the five combinations were selected for subsequent mechanistic research.

#### 3.1.4 Effects of TDS combinations on LDH levels in glu-induced cell injury model

**FIGURE 3:**
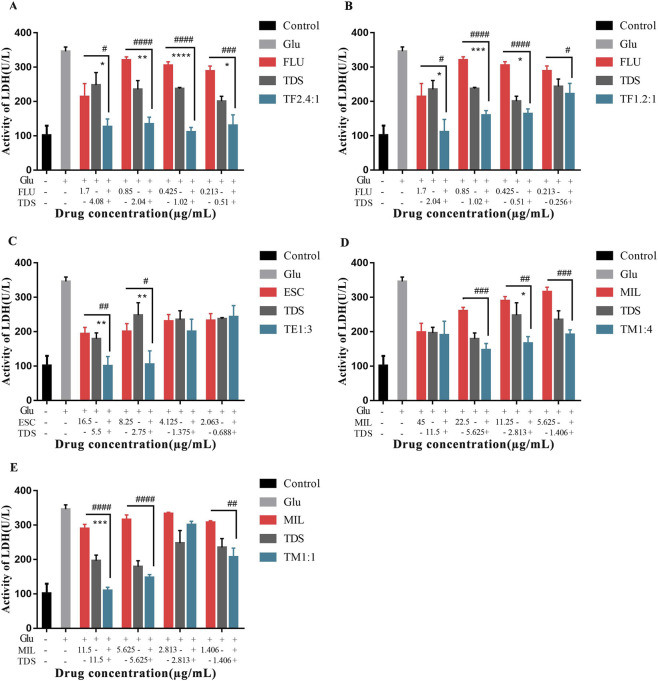
The LDH activity of TF2.4:1 **(A)** and 1.2:1 **(B)** on Glu-induced PC12 injured cell, the LDH activity of TE1:3 **(C)** on Glu-induced PC12 injured cell, and the LDH activity of TM1:4 **(D)** and 1:1 **(E)** on Glu-induced PC12 injured cell. Compared with TDS group: *P < 0.05, **P < 0.01, ***P < 0.001, ***P < 0.0001, compared with FLU, ESC and MIL groups: #P < 0.05, ##P < 0.01, ###P < 0.001, ####P < 0.0001.

The LDH assay was further performed to verify the membrane protective effects of TDS combinations with different ratios on glutamate-injured PC12 cells, and the results are presented in Figure 3. Compared with the normal control group, LDH activity was markedly increased in the 10 mM glutamate model group (P < 0.0001), indicating that glutamate exposure caused severe cell membrane damage and intracellular content leakage, which verified the successful establishment of the PC12 cell injury model.

For TF combinations (TDS: FLU), all concentration groups of the 2.4:1 and 1.2:1 ratios exhibited significantly lower LDH activity than the corresponding TDS and FLU monotherapy groups, demonstrating that TF combination treatment effectively alleviated glutamate-mediated cell membrane injury. For the TE combination (TDS: ESC, 1:3), no significant difference in LDH levels was observed between the combined group and single-drug groups at low concentrations, suggesting insufficient stability of the synergistic protective effect of this ratio. In the TM combinations (TDS:MIL, 1:4 and 1:1), LDH activity in several combination groups showed no significant difference or even slightly higher levels relative to monotherapy groups, revealing certain limitations in the synergistic protection of TM compound formulations.

Combined with the data from MTT cell viability assays and LDH detection, TF combinations (1.2:1 and 2.4:1) steadily improved the viability of glutamate-damaged cells and markedly reduced LDH release, showing the most potent and stable synergistic neuroprotective effects. Consequently, TF-1 (TDS: FLU = 1.2:1, w/w) and TF-2 (TDS: FLU = 2.4:1, w/w) were selected for subsequent *in vivo* pharmacodynamic and mechanistic investigations.

### 3.2 *In vivo* pharmacokinetic study of TF-1 and TF-2

**FIGURE 4:**
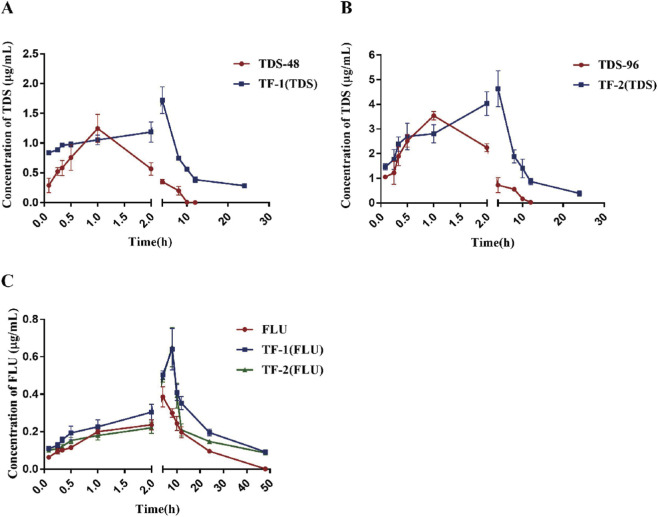
Plasma concentration-time curves of TDS and FLU in rats after oral administration. **(A)** Plasma concentration-time curve of TDS after oral administration of TDS and TF-1 in rats. **(B)** Plasma concentration-time curve of TDS after oral administration of TDS and TF-2 in rats. **(C)** Plasma concentration-time curve of FLU after oral administration of FLU, TF-1 and TF-2 in rats. Data are expressed as mean ± standard deviation (x ® ± s), n = 5.

To investigate the potential pharmacokinetic interaction between TDS and FLU following combined administration, blood drug concentrations in rats at different time points were determined after intragastric administration of TDS monotherapy (48 and 96 mg/kg), FLU monotherapy (40 mg/kg), and TF combinations (TF-1: 48 mg/kg TDS +40 mg/kg FLU; TF-2: 96 mg/kg TDS +40 mg/kg FLU). The plasma concentration–time curves were plotted (Figure 4), and major pharmacokinetic parameters were calculated using DAS 2.1.1 software (Tables 3, 4).

**TABLE 3:** The pharmacokinetic parameters of TDS after oral administration of different doses of TDS, TF-1 and TF-2.

Parameter	Unit	TDS-48 (mg/L)	TF-1 (mg/L)	TDS-96 (mg/L)	TF-2 (mg/L)
AUC(0-t)	mg/l*h	3.914	16.200	11.613	40.594
MRT (0-t)	h	3.087	7.921	3.078	6.746
t_1/2_z	h	1.224	3.719	1.505	3.359
Tmax	h	1	4	1	4
Cmax	mg/L	1.250	1.722	3.540	4.631
Vz	L/kg	21.635	15.684	17.878	11.360
CLz	L/h/kg	12.254	2.923	8.235	2.344

**TABLE 4:** The pharmacokinetic parameters of FLU after oral administration of FLU, TF-1 and TF-2.

Parameter	Unit	FLU (mg/L)	TF-1 (mg/L)	TF-2 (mg/L)
AUC(0-t)	mg/l*h	5.063	12.012	9.916
MRT (0-t)	h	9.449	16.211	16.272
t_1/2_z	h	6.563	17.346	28.888
Tmax	h	4	8	8
Cmax	mg/L	0.386	0.640	0.652
Vz	L/kg	67.815	70.580	123.542
CLz	L/h/kg	7.140	2.820	2.964

#### 3.2.1 Pharmacokinetic characteristics of TDS

After intragastric administration in rats, TDS was rapidly absorbed *in vivo* (Figures 4A,B). Compared with the TDS monotherapy groups at corresponding doses, the plasma concentrations of TDS in the TF-1 and TF-2 groups were significantly increased after 1 h, with a slower elimination rate, and relatively high drug concentrations were still detectable at 24 h.

Pharmacokinetic parameters (Table 3) showed that, compared with the TDS monotherapy groups, the exposure of TDS was significantly increased and its elimination was markedly delayed in the TF combination groups. For AUC_(0-t)_: the AUC_(0-t)_ of TDS in the TF-1 group was 16.200 mg/Lh, which was 4.139-fold higher than that in the TDS-48 monotherapy group (3.914 mg/Lh); the AUC_(0-t)_ of TDS in the TF-2 group was 40.594 mg/Lh, which was 3.527-fold higher than that in the TDS-96 monotherapy group (11.613 mg/Lh).

For half-life (t_1_/_2_z): the t_1_/_2_z of TDS in the TF-1 group was 3.719 h, 3.04-fold longer than that in the TDS-48 group (1.224 h); the t_1_/_2_z of TDS in the TF-2 group was 3.359 h, 2.23-fold longer than that in the TDS-96 group (1.505 h). For mean residence time (MRT_(0-t)_): the MRT_(0-t)_ of TDS in the TF-1 and TF-2 groups were 7.921 h and 6.746 h, respectively, which were 2–3-fold longer than those in the corresponding monotherapy groups (3.087 h and 3.078 h).

For clearance (CLz): the CLz values of TDS in the TF-1 and TF-2 groups were 2.923 L/h/kg and 2.344 L/h/kg, respectively, which were significantly lower than those in the corresponding monotherapy groups (12.254 L/h/kg and 8.235 L/h/kg). For peak concentration (Cmax): the Cmax values of TDS in the TF-1 and TF-2 groups were 1.722 mg/L and 4.631 mg/L, respectively, which were higher than those in the corresponding monotherapy groups (1.250 mg/L and 3.540 mg/L). For time to peak (Tmax): the Tmax of TDS in both TF-1 and TF-2 groups was 4 h, which was markedly prolonged compared with the monotherapy groups (1 h).

#### 3.2.2 Pharmacokinetic characteristics of FLU

Compared with the FLU monotherapy group, the plasma FLU concentrations at each time point were significantly increased, drug elimination was delayed, and the area under the concentration–time curve was markedly enlarged in the TF-1 and TF-2 groups (Figure 4C).

As summarized in Table 4, the exposure of FLU was significantly increased and its elimination was markedly delayed in the TF combination groups relative to the FLU monotherapy group. For AUC_(0-t)_: the AUC_(0-t)_ of FLU in the TF-1 group was 12.012 mg/Lh, which was 2.373-fold higher than that in the FLU monotherapy group (5.063 mg/Lh); the AUC_(0-t)_ of FLU in the TF-2 group was 9.916 mg/Lh, which was 2.00-fold higher than that in the FLU monotherapy group.

For half-life (t_1_/_2_z): the t_1_/_2_z of FLU in the TF-1 group was 17.346 h, 2.643-fold longer than that in the FLU monotherapy group (6.563 h); the t_1_/_2_z of FLU in the TF-2 group was 28.888 h, 4.402-fold longer than that in the FLU monotherapy group. For mean residence time (MRT_(0-t)_): the MRT_(0-t)_ of FLU in the TF-1 and TF-2 groups were 16.211 h and 16.272 h, respectively, both approximately 1.7-fold longer than that in the FLU monotherapy group (9.449 h).

For clearance (CLz): the CLz values of FLU in the TF-1 and TF-2 groups were 2.820 L/h/kg and 2.964 L/h/kg, respectively, which were significantly lower than that in the FLU monotherapy group (7.140 L/h/kg). For peak concentration (Cmax): the Cmax values of FLU in the TF-1 and TF-2 groups were 0.640 mg/L and 0.652 mg/L, respectively, which were higher than that in the FLU monotherapy group (0.386 mg/L). For time to peak (Tmax): the Tmax of FLU in both TF-1 and TF-2 groups was 8 h, which was markedly prolonged compared with the FLU monotherapy group (4 h).

In summary, compared with the monotherapy groups, the AUC values of TDS and FLU were significantly increased, t_1_/_2_z and MRT were markedly prolonged, and CLz was significantly decreased in the TF-1 and TF-2 combination groups.

### 3.3 Anti-anxiety and anti-depressive effects and mechanism research of TF-1 and TF-2

#### 3.3.1 Evaluation of the comorbid anxiety and depression (CAD) model

**FIGURE 5:**
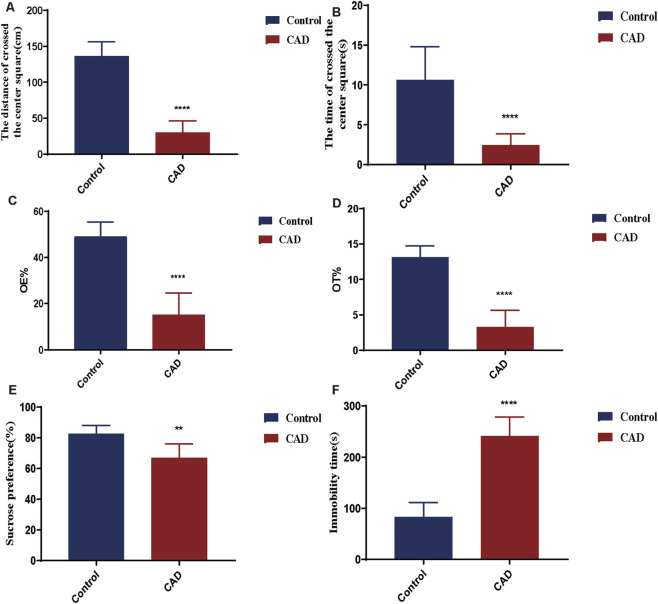
**(A)** Movement distance in the central region of normal control and CAD group mice. **(B)** Activity time in the central region of the mice in the normal control group and CAD group. **(C)** Percentage of mice in normal control and CAD groups entering the arm opening. **(D)** Percentage of mice in normal control and CAD groups entering the arm opening. **(E)** Sucrose predisposition in normal control and CAD mice. **(F)** The immobility time of the mice in the normal control group and the CAD group, compared with the normal control group: **P < 0.01, ****P < 0.0001.

To verify whether the chronic unpredictable mild stress (CUMS) combined with single-housing regimen successfully induced the CAD model, behavioral assessments were conducted in mice of each group through the open field test (OFT), elevated plus maze (EPM), sucrose preference test (SPT) and forced swimming test (FST). The results are shown in Figure 5.

The OFT results revealed that, compared with the normal control group, the traveling distance and residence time in the central area of the open field were significantly reduced in the CAD model group (P < 0.0001), suggesting decreased spontaneous exploration ability and obvious anxiety-like behaviors. The EPM results demonstrated that the open arm entry percentage (OE%) and open arm time percentage (OT%) in the CAD model group were markedly lower than those in the normal control group (P < 0.0001), indicating intensified fear of open environments and severe anxiety-like performance.

In the SPT, the sucrose preference rate of CAD model mice was significantly decreased (P < 0.01), which reflected anhedonia and other typical depressive-like behaviors. In the FST, the cumulative immobility time was distinctly prolonged in the CAD model group (P < 0.0001), presenting behavioral despair characteristic of depression.

In addition, mice in the CAD model group exhibited obvious abnormal physiological manifestations relative to normal mice, including messy fur, listlessness and decreased voluntary activity. Collectively, mice in the CAD group displayed remarkable anxiety- and depressive-like alterations in OFT, EPM, SPT and FST. It was confirmed that the CAD mouse model was successfully established, which could be applied to subsequent pharmacodynamic evaluation.

#### 3.3.2 Behavioral effects of TF-1 and TF-2 on CAD model mice

##### 3.3.2.1 Results of OFT and EPM

**FIGURE 6:**
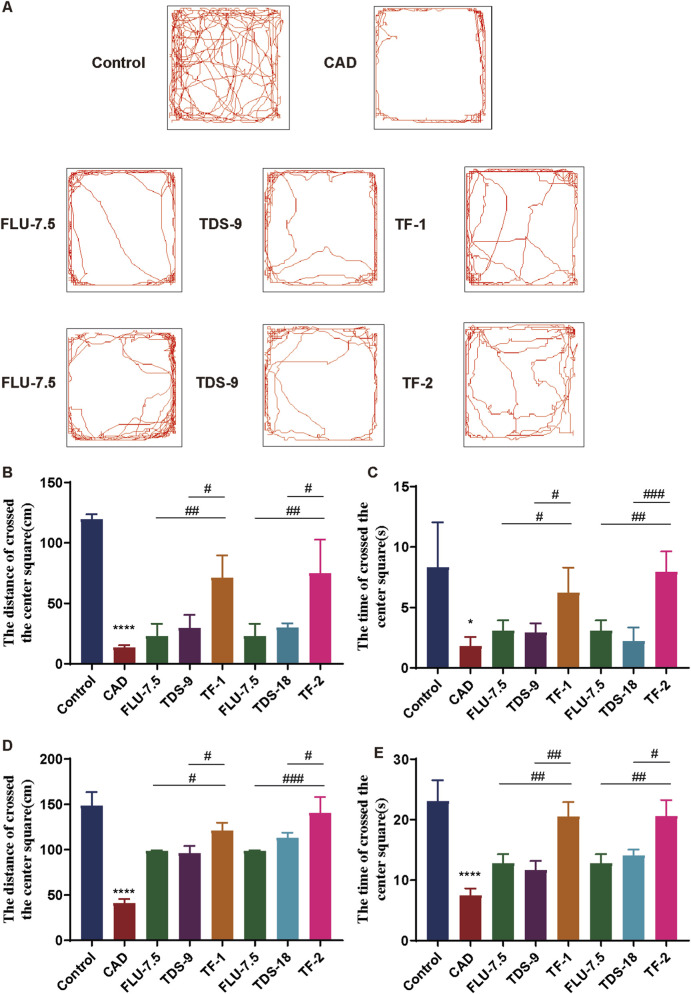
OFT evaluation results of mice after 28 and 49 days of administration **(A)** The activity track diagram **(B,D)** The activity distance in the central area of mice after 28 and 49 days of administration **(C,E)** The activity time in the central area of mice after 28 and 49 days of administration. Compared with the normal control group: *P < 0.05, ****P < 0.0001, compared with the single group: ^#^P < 0.05, ^##^P < 0.01, ^###^P < 0.001.

**FIGURE 7:**
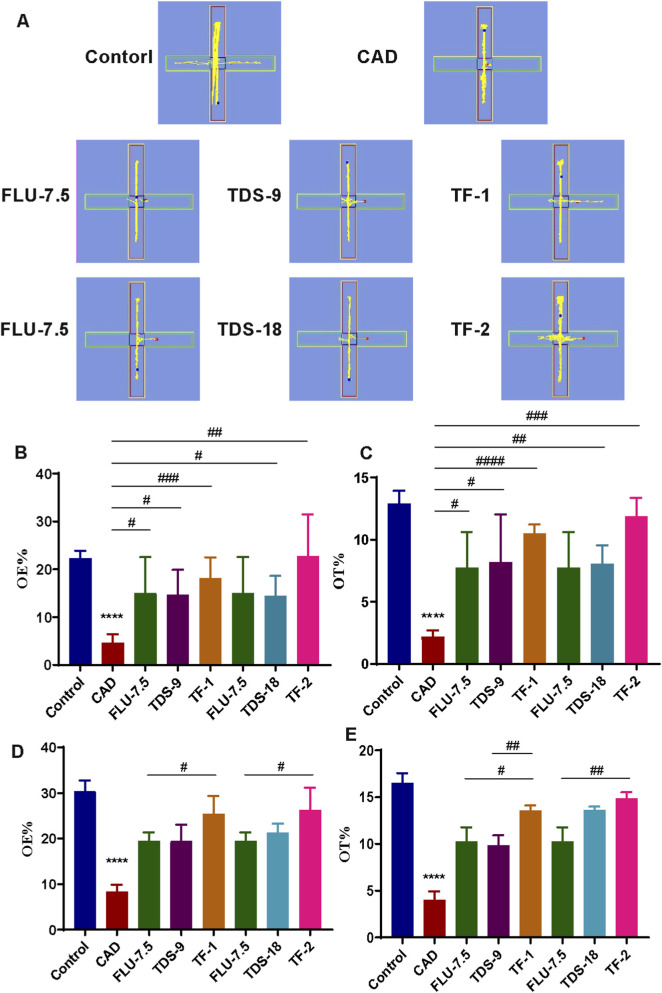
EPM evaluation results of mice in each group after 28 and 49 days of administration **(A)** activity track diagram **(B,D)** OE% of mice after 28 and 49 days of administration **(C,E)** OT% of mice after 28 and 49 days of administration. Compared with the normal control group: ****P < 0.0001, compared with CAD or single group: ^#^P < 0.05, ^##^P < 0.01, ^###^P < 0.001, ^####^P < 0.0001.

To evaluate the ameliorative effects of TF-1 and TF-2 on comorbid anxiety and depression in CAD mice, the OFT and EPM were performed after 28-day and 49-day administration, respectively. The results are shown in Figures 6, 7.

1. Results of OFT

After 28 days of administration (Figure 6), compared with the CAD model group, the central traveling distance and residence time of mice in all drug-treated groups were increased to varying degrees. Compared with the TDS monotherapy groups (9, 18 mg/kg) and FLU monotherapy group (7.5 mg/kg):

In the TF-1 group, the central traveling distance was 71.23 ± 18.41 cm, which was significantly higher than that in the TDS-9 group (29.83 ± 5.35 cm) and FLU group (34.63 ± 5.15 cm) (P < 0.05), with an increase of approximately 100%. The central residence time was 6.25 ± 2.06 s, also markedly longer than that in the TDS-9 group (3.08 ± 0.84 s) and FLU group (2.94 ± 0.75 s) (P < 0.05), rising by nearly 100%.

In the TF-2 group, the central traveling distance was 74.93 ± 27.77 cm, significantly higher than that in the TDS-18 group (29.83 ± 5.35 cm) and FLU group (29.97 ± 3.55 cm) (P < 0.05), with an increase of about 100%. The central residence time was 7.96 ± 1.66 s, which was remarkably prolonged compared with the TDS-18 group (3.08 ± 0.84 s) and FLU group (2.21 ± 1.13 s) (P < 0.01), with a 100% elevation.

After 49 days of continuous treatment, the beneficial effects of TF combinations on spontaneous activity were further enhanced. Compared with the 28-day intervention:

In the TF-1 group, the central traveling distance increased from 71.23 ± 18.41 cm to 121.13 ± 8.85 cm (P < 0.05), an increase of approximately 80%; the central residence time extended from 6.25 ± 2.06 s to 20.50 ± 2.41 s (P < 0.01), with an increase of 158%.

In the TF-2 group, the central traveling distance increased from 74.93 ± 27.77 cm to 140.87 ± 17.32 cm (P < 0.05, ∼80% increase); the central residence time increased from 7.96 ± 1.66 s to 20.58 ± 2.69 s (P < 0.01, ∼158% increase).

2. Results of EPM

After 28 days of administration (Figure 7), the open arm entry percentage (OE%) and open arm time percentage (OT%) were significantly elevated in all drug intervention groups relative to the CAD model group. Compared with monotherapy groups, OE% and OT% were moderately increased in the TF-1 and TF-2 groups, without significant statistical difference.

After 49 days of treatment, the anxiolytic effects of TF combinations were more prominent. Compared with each single-drug group:

The TF-1 group presented an OE% of 25.38% ± 4.00%, significantly higher than that in the monotherapy group (19.51% ± 1.87%) (P < 0.05); its OT% was 13.59% ± 0.51%, which was also markedly higher than that in the monotherapy group (10.27% ± 1.52%) (P < 0.05).

The TF-2 group showed an OE% of 26.27% ± 4.96%, significantly higher than the monotherapy group (19.51% ± 1.87%) (P < 0.05); its OT% was 14.85% ± 0.67%, significantly higher than that in the monotherapy group (9.87% ± 1.05%) (P < 0.05).

Collectively, combined with the OFT and EPM results, 49-day continuous administration of TF-1 and TF-2 could notably alleviate anxiety-like behaviors in CAD mice, with superior efficacy to monotherapy at corresponding doses.

##### 3.3.2.2 Results of SPT and FST

**FIGURE 8:**
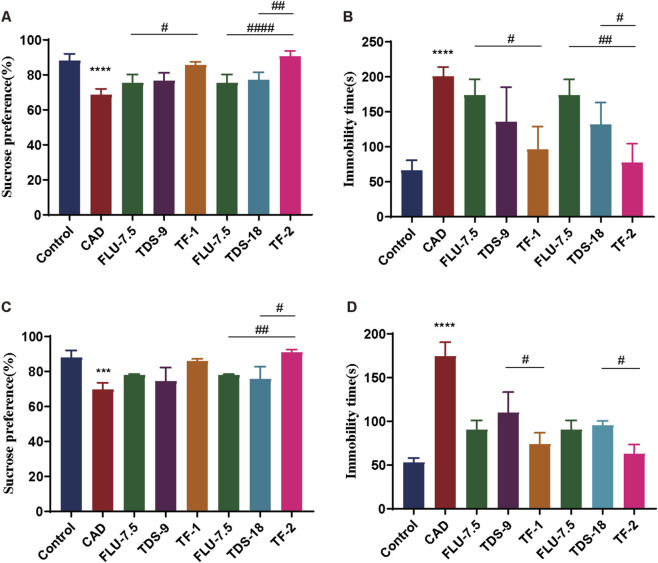
**(A,C)** The sucrose preference of mice after 28 and 49 days of administration **(B,D)** The immobility time of mice after 28 and 49 days of administration. Compared with the normal control group: ***P < 0.001, ****P < 0.0001, compared with the single group: ^#^P < 0.05, ^##^P < 0.01, ^####^P < 0.0001.

To evaluate the ameliorative effects of TF-1 and TF-2 on depressive-like behaviors in mice with CUMS-induced comorbid anxiety and depression, the SPT and FST were conducted after 28 and 49 days of administration. The results are presented in Figure 8.

1. Results of SPT

The SPT was applied to assess the degree of anhedonia in mice, and the relevant data are shown in Figures 8A,C. After 28 days of treatment, the sucrose preference rate was significantly increased in all drug-intervened groups compared with the CAD model group. In comparison with the corresponding monotherapy groups, the TF-1 group exhibited a markedly higher sucrose preference rate than the TDS-9 and FLU-7.5 groups (P < 0.05); similarly, the TF-2 group showed significantly elevated sucrose preference relative to the TDS-18 and FLU-7.5 groups (P < 0.05). After 49 days of continuous administration, the therapeutic effects of TF combinations on anhedonia were further strengthened. The sucrose preference in the TF-1 group was significantly higher than that in the CAD model group (P < 0.01). The TF-2 group displayed a significant increase in sucrose preference when compared with the TDS-18 and FLU-7.5 monotherapy groups (P < 0.05), as well as the CAD model group (P < 0.01).

2. Results of FST

The FST was used to evaluate the degree of behavioral despair, and the results are shown in Figures 8B,D. After 28 days of administration, the immobility time of mice in each treatment group was reduced to varying degrees relative to the CAD model group. Compared with matched single-drug groups, the TF-1 group had distinctly shorter immobility time than the FLU-7.5 group (P < 0.05), while the TF-2 group showed a significant reduction in immobility time versus the TDS-18 and FLU-7.5 groups (P < 0.05). Following 49-day intervention, the antidepressant efficacy of TF combinations was further enhanced. The immobility time in the TF-1 group was significantly lower than that in the TDS-9 and FLU-7.5 monotherapy groups (P < 0.05), and the TF-2 group also presented obviously decreased immobility time compared with the TDS-18 and FLU-7.5 monotherapy groups (P < 0.05). During behavioral testing, mice in the drug administration groups also showed improved general conditions, including increased food intake and enhanced voluntary activity, in contrast to CAD model mice.

In summary, based on the SPT and FST findings, long-term treatment for 49 days enabled both TF-1 and TF-2 to effectively ameliorate depressive-like behaviors (anhedonia and behavioral despair) in CAD mice, with superior efficacy compared to monotherapy at equivalent doses.

#### 3.3.3 Effects of TF-1 and TF-2 on 5-HT and BDNF contents in plasma and hippocampus of CAD mice

**FIGURE 9:**
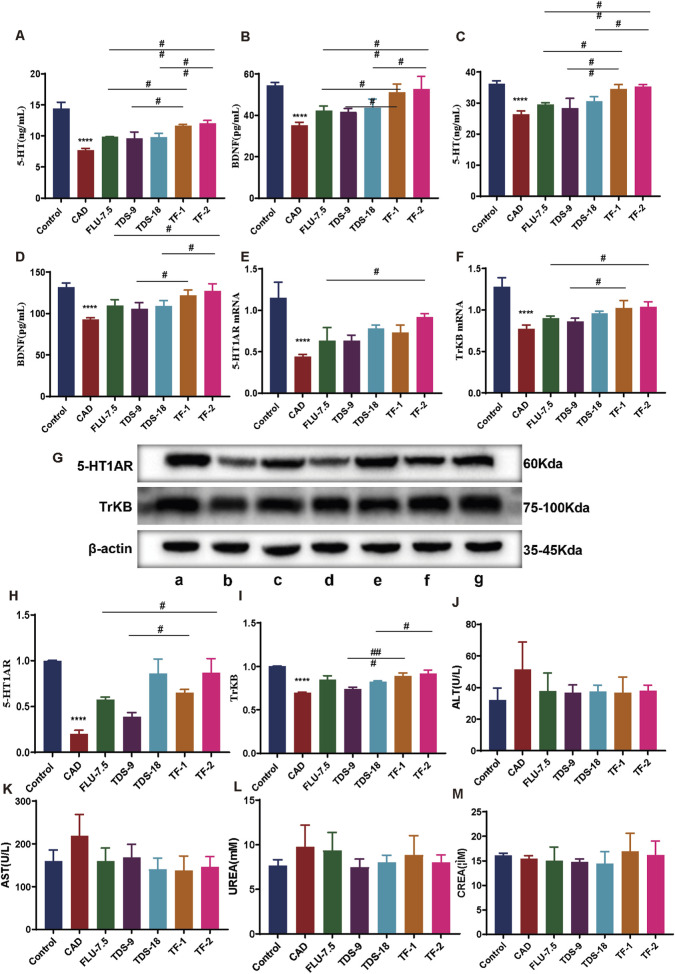
**(A–D)** The content of 5-HT **(A)** and BDNF **(B)** in the plasma of mice and the content of 5-HT **(C)** and BDNF **(D)** in the hippocampus. Compared with the normal control group: ****P < 0.0001, compared with the single-agent group: #P < 0.05, ##P < 0.01. **(E,F)** expression levels of 5-HT1AR mRNA **(E)** and TrKB mRNA **(F)** in mouse hippocampal tissue. Compared with normal control group: ****P < 0.0001; compared with single drug group: #P < 0.05. **(G–I)** Expression levels of 5-HT1AR and TrKB proteins in hippocampal tissues of mice **(G)** 5-HT1AR, TrKB and β-actin Western blot bands, a, normal control group; b, CAD group; c, FLU-7.5 group; d, TDS-9 group; e, TDS-18 group; f, TF-1 group; and g, TF-2 group. Compared with the normal control group: ****P < 0.0001; compared with the single-drug group: #P < 0.05, ###P < 0.001 **(J–M)** changes in biochemical indicators related to liver and kidney function in mice **(J,K)** Changes in plasma ALT and AST contents in mice **(L,M)** Changes in plasma CREA and UREA contents in mice.

To preliminarily explore the underlying mechanisms of TF-1 and TF-2 against anxiety and depression, the concentrations of serotonin (5-HT) and brain-derived neurotrophic factor (BDNF) in mouse plasma and hippocampal tissues of each group were determined by ELISA after behavioral evaluation at 28 days of administration. The results are shown in Figures 9A–D. Compared with the normal control group, the levels of 5-HT and BDNF in plasma and hippocampus were significantly downregulated in the CAD model group (P < 0.05 or P < 0.01), indicating that the CAD model successfully induced disorders of monoamine neurotransmitters and neurotrophic factors in mice.

After 28 days of treatment, the contents of 5-HT and BDNF in the FLU-7.5, TDS-9 and TDS-18 monotherapy groups were slightly increased relative to the CAD model group, without significant statistical differences (P > 0.05). In contrast, the levels of 5-HT and BDNF in plasma and hippocampus were markedly elevated in the TF-1 and TF-2 groups compared with all monotherapy groups (P < 0.05). Both TF-1 and TF-2 significantly increased 5-HT contents in plasma and hippocampus when compared with TDS and FLU monotherapy at corresponding doses. Similarly, the BDNF levels in plasma and hippocampus were also remarkably higher in the two combined groups.

In conclusion, the 5-HT and BDNF systems were functionally impaired in CAD model mice. After 28 consecutive days of administration, TF-1 and TF-2 significantly upregulated the expression of 5-HT and BDNF in plasma and hippocampal tissues, exerting superior effects to single treatment with FLU or TDS.

#### 3.3.4 Effects of TF-1 and TF-2 on the expression of 5-HT_1_AR and TrKB in the hippocampus of CAD mice

Quantitative real-time PCR (qPCR) and Western blot (WB) assays were used to detect the mRNA and protein expression levels of 5-HT_1_AR and TrKB in the hippocampus, so as to further explore the molecular mechanisms underlying the anxiolytic and antidepressant effects of TF-1 and TF-2. The results are presented in Table 5, Figures 9E–I.

**TABLE 5:** Relative mRNA and protein expression of 5-HT1AR and TrKB in the hippocampus of mice. ( $\bar{x}$ ± s, n = 3).

Detection type	Group	5-HT1AR	TrKB
mRNA	Normal control	1.15 ± 0.19	1.27 ± 0.11
	CAD	0.44 ± 0.02	0.77 ± 0.04
	FLU-7.5	0.63 ± 0.15	0.90 ± 0.03
	TDS-9	0.63 ± 0.07	0.86 ± 0.04
	TDS-18	0.78 ± 0.04	0.96 ± 0.02
	TF-1	0.73 ± 0.19	1.02 ± 0.09
	TF-2	0.92 ± 0.04	1.04 ± 0.06
Protein	Normal control	1.00 ± 0.04	1.00 ± 0.05
	CAD	0.20 ± 0.04	0.69 ± 0.01
	FLU-7.5	0.58 ± 0.02	0.85 ± 0.04
	TDS-9	0.39 ± 0.05	0.74 ± 0.02
	TDS-18	0.86 ± 0.16	0.82 ± 0.01
	TF-1	0.65 ± 0.03	0.89 ± 0.03
	TF-2	0.87 ± 0.16	0.92 ± 0.04

Compared with the normal control group, the mRNA and protein expression of 5-HT_1_AR and TrKB were significantly downregulated in the CUMS-CAD model group (P < 0.05 or P < 0.01), indicating that CUMS-CAD intervention markedly inhibited the expression of key receptors in the hippocampus. Relative to the CUMS-CAD model group, all drug treatment groups exhibited significant upregulation of hippocampal 5-HT_1_AR and TrKB mRNA expression (P < 0.05 or P < 0.01). In terms of 5-HT_1_AR mRNA levels, TF-1 and TF-2 exerted stronger upregulatory effects than TDS and FLU monotherapy at corresponding doses. The TrKB mRNA levels in TF combination groups were significantly higher than those in all monotherapy groups (P < 0.05).

Meanwhile, the protein expression of 5-HT_1_AR and TrKB was effectively increased in each administration group (P < 0.05 or P < 0.01). The protein levels of 5-HT_1_AR in the TF-1 and TF-2 groups were higher than those in the TDS and FLU monotherapy groups, and TrKB protein expression was markedly elevated in the combination groups compared with single-drug treatment (P < 0.05), suggesting that TF combinations possessed a prominent superiority in upregulating TrKB protein.

In summary, the hippocampal tissues of CUMS-CAD model mice showed low expression of 5-HT_1_AR and TrKB. TF-1 and TF-2 could significantly increase the mRNA and protein levels of both targets, with better overall regulatory effects than TDS and FLU monotherapy. In particular, the compound formulations displayed distinct advantages in promoting TrKB expression.

#### 3.3.5 Effects of TF-1 and TF-2 on hepatic and renal functions in CAD mice

To evaluate the safety of long-term administration of TF-1 and TF-2 as well as their restorative effects on CAD-induced hepatic and renal dysfunction, the plasma levels of alanine transaminase (ALT), aspartate transaminase (AST), creatinine (CREA) and urea (UREA) were detected after 49 days of treatment. The results are shown in Table 6, Figures 9J–M.

**TABLE 6:** The related biochemical indexes of liver and kidney function in mice ( $\bar{x}$ ± s, n = 3).

Group	ALT (U/L)	AST (U/L)	CREA (µM)	UREA (mM)
Normal control	32.07 ± 7.57	159.60 ± 26.31	16.10 ± 0.50	7.63 ± 0.71
CAD	51.53 ± 17.26	219.00 ± 49.46	15.40 ± 0.66	9.74 ± 2.47
FLU-7.5	37.70 ± 11.54	159.77 ± 30.47	15.03 ± 2.80	9.35 ± 2.03
TDS-9	36.67 ± 4.95	168.73 ± 30.63	14.80 ± 0.61	7.47 ± 0.91
TDS-18	37.37 ± 4.21	140.33 ± 26.48	14.40 ± 2.51	8.02 ± 0.82
TF-1	36.63 ± 9.99	137.43 ± 34.25	16.93 ± 5.70	8.83 ± 2.20
TF-2	38.10 ± 3.41	146.00 ± 24.30	16.17 ± 4.88	8.01 ± 0.85

Compared with the normal control group, the CAD model group presented significantly elevated ALT and AST levels (P < 0.05), while no remarkable differences were observed in CREA and UREA contents (P > 0.05). In all drug-intervened groups (FLU-7.5, TDS-9, TDS-18, TF-1 and TF-2), ALT and AST levels showed no significant changes relative to the normal control group (P > 0.05). Hepatic function indicators in each treatment group declined to varying degrees when compared with the CAD model group. Notably, TF-1 and TF-2 exerted the most prominent ameliorative effects, with indicators returning close to normal levels, indicating that TF combinations could alleviate CAD-mediated hepatic injury. No significant differences in renal function parameters were found among all administration groups and the normal control group (P > 0.05), and no obvious renal abnormalities were observed.

In conclusion, chronic CAD stress obviously induced hepatic dysfunction in mice, whereas it caused negligible damage to renal function. Long-term administration of all tested drugs exhibited no overt hepatotoxicity or nephrotoxicity. TF-1 and TF-2 could effectively repair CAD-caused hepatic damage and restore liver function indicators to the normal range, demonstrating favorable *in vivo* safety at therapeutic doses.

#### 3.3.6 Effects of TF-1 and TF-2 on the pathological morphology of hippocampus, heart, liver and kidney tissues in mice

**FIGURE 10:**
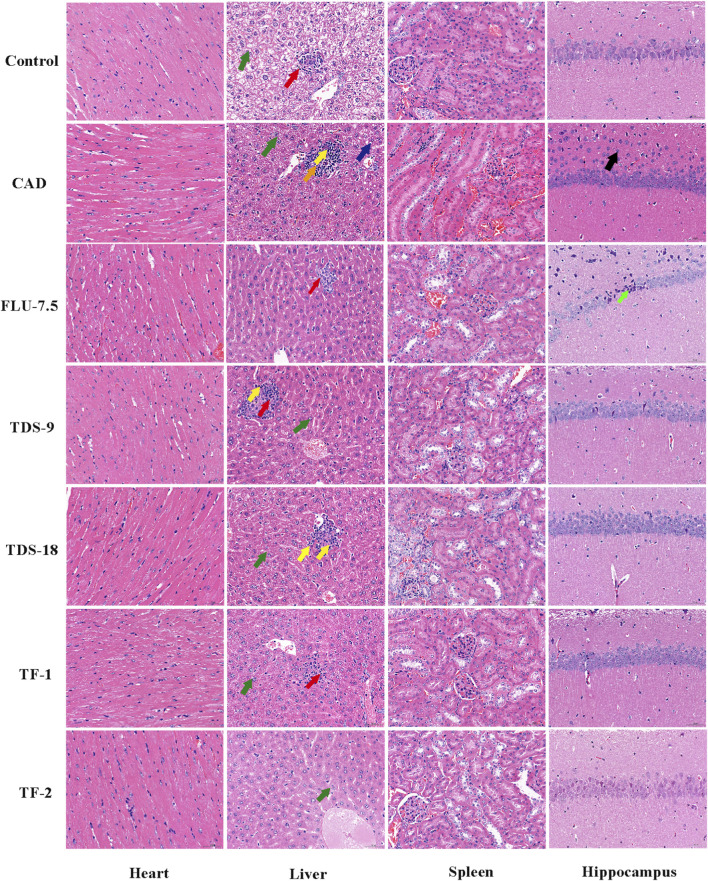
HE staining of heart, liver, kidney and hippocampus of mice (×400).

To evaluate the effects of long-term administration of TF-1 and TF-2 on visceral histomorphology in CUMS-CAD mice, hippocampal, cardiac, hepatic and renal tissues were collected for HE staining after 49 consecutive days of treatment, and pathological changes were observed under a light microscope (Figure 10).

Hippocampus: In the normal control group, pyramidal cells in the hippocampal CA1 region were neatly and densely arranged with intact cell morphology and clear nucleoli. Compared with the normal control group, pyramidal cells in the CUMS-CAD model group were loosely arranged, accompanied by karyopyknosis and hyperchromatism in a small number of cells. In all drug-treated groups, the hippocampal structure remained intact with regular cell arrangement, no obvious gliosis or inflammatory infiltration, and the histological morphology was basically consistent with that of the normal group.

Liver: The normal liver presented complete hepatic lobule structure and regularly arranged hepatocytes, without degeneration, necrosis or inflammatory infiltration. In the CUMS-CAD model group, punctate necrosis and inflammatory cell infiltration were observed in hepatocytes. Compared with the model group, only mild hepatic pathological damage was found in each administration group, mainly manifested as hepatocellular vacuolar degeneration, punctate necrosis and slight inflammatory infiltration. No significant differences in hepatic lesions were observed between the TF-1/TF-2 groups and TDS/FLU monotherapy groups, with no aggravation of tissue injury.

Heart: In the normal control group, myocardial fibers were orderly arranged with distinct striations and no interstitial inflammatory infiltration. No obvious myocardial pathological abnormalities were detected in either the CUMS-CAD model group or each drug intervention group.

Kidney: The normal kidney showed intact glomerular and tubular structures with regular epithelial arrangement. Renal morphology in the model group and all administration groups remained generally normal, without obvious organic damage.

In summary, CUMS-CAD stress induced mild hepatic pathological damage, mainly characterized by punctate necrosis and inflammatory infiltration, while causing slight injuries to the hippocampus, heart and kidney. Long-term treatment with TF-1 and TF-2 did not exacerbate visceral pathological lesions, and the degree of hepatic injury was comparable to that of monotherapy. These findings indicated that TF-1 and TF-2 exerted no obvious toxic effects on major vital organs of CUMS-CAD mice at therapeutic doses, presenting satisfactory *in vivo* safety.

## 4 Discussion

Comorbid anxiety and depression (CAD) represents a highly prevalent subtype of mental disorders globally, which is characterized by a chronic clinical course, poor treatment responsiveness, and a high risk of recurrence. Meanwhile, it is accompanied by elevated suicide risk and excessive consumption of medical resources, which has become a crucial public health concern (Virtanen et al., 2020; Zhai et al., 2025). With the development of precision psychiatry, the clinical therapeutic goals for CAD have shifted from simple symptom control to strategies with rapid onset, long-lasting efficacy and milder adverse reactions (Tusa et al., 2025; Fairweather et al., 2026). At present, the clinical response rate of first-line monotherapies, such as selective serotonin reuptake inhibitors and serotonin-norepinephrine reuptake inhibitors, is only 40%–60% in CAD treatment. These agents also present a slow therapeutic onset and potential risks of disease deterioration (Kato et al., 2021; Gurbanov et al., 2023; Gheysens et al., 2024; Vázquez and Baldessarini, 2025). Therefore, combined administration of anxiolytics and antidepressants has become a vital strategy to overcome the limitations of conventional CAD treatment. Nevertheless, unreasonable drug combination may trigger drug–drug interactions, drug tolerance and treatment resistance, which severely restrict its clinical popularization and application (Dos Santos et al., 2019; Kendrick, 2021). Accordingly, screening rational drug combinations with synergistic effects, definite mechanisms and favorable safety has become the core research focus and breakthrough point in the field of CAD therapy (Hampf et al., 2023).

The pathogenesis of CAD is multifactorial and complicated, involving neurotransmitter imbalance, impaired neural plasticity, hypothalamic–pituitary–adrenal (HPA) axis dysfunction and chronic stress (Knowles and Olatunji, 2020; Ferrer et al., 2026). Among these pathological changes, dysfunction of the serotonin (5-hydroxytryptamine, 5-HT) system acts as a core pathological link, directly affecting disease progression and therapeutic outcomes (Maes et al., 2025). As a highly selective 5-HT_1_A receptor agonist, tandospirone (TDS) regulates synaptic 5-HT levels and exerts prominent anxiolytic and antidepressant effects, which makes it an ideal adjuvant agent for antidepressants (Antypa et al., 2016; International, 2024). Accumulating clinical evidence has demonstrated that the combined use of TDS and SSRIs can significantly accelerate the reduction of Hamilton Anxiety Scale (HAMA) and Hamilton Depression Scale (HAMD) scores in CAD patients, accompanied by a decreased incidence of adverse reactions (Demyttenaere et al., 2021). Basic studies have also confirmed that such combination therapy can ameliorate the hypofunction of the 5-HTergic system by downregulating the density of hippocampal 5-HT_1_A autoreceptors (Bandoli et al., 2017). In addition, antidepressants with different molecular targets, including fluoxetine (FLU), escitalopram (ESC) and milnacipran (MIL), possess respective pharmacological advantages (Yang et al., 2020; Li S. et al., 2023; 2025). However, their monotherapeutic efficacy against CAD is limited by single-target regulation defects (Butkevich et al., 2019; Belus et al., 2022), and there is still no standardized and widely applicable combined medication regimen for clinical practice (Wang and Tu, 2026).

Based on the above clinical demands and theoretical foundations, this study innovatively constructed a novel combined medication system of tandospirone citrate with three representative antidepressants, namely, TF (TDS-FLU), TE (TDS-ESC) and TM (TDS-MIL), in which TDS was separately combined with fluoxetine (FLU, SSRIs), escitalopram (ESC, high-selective SSRIs) and milnacipran (MIL, SNRIs).

In the *in vitro* experiment, referring to the toxicity classification standards of the United States Pharmacopeia (Kilic et al., 2023; USP, 2010), the MTT assay was adopted to determine the maximum safe concentration and optimal detection time of each single drug in PC12 cells, thereby establishing a standardized experimental system. Hippocampal neuronal damage is one of the core pathological features of CAD, manifested as neuronal atrophy, apoptosis and decreased synaptic density. The therapeutic efficacy of antidepressants is closely correlated with their neuroprotective properties (Camargos et al., 2020; Xu et al., 2022). Excessive release of the excitatory amino acid glutamate (Glu) induces excitotoxicity, which is a key driver of hippocampal neuronal injury. Glutamate exposure triggers calcium influx, reactive oxygen species (ROS) accumulation and lipid peroxidation, ultimately leading to neuronal apoptosis (Zheng et al., 2021). In the present study, a Glu-induced PC12 cell injury model was established based on this mechanism. The results revealed that 2 TF ratios (TF-1 and TF-2) exerted superior effects in improving the viability of injured cells compared with TE, TM and monotherapy groups, verifying the synergistic neuroprotective effect of TDS combined with FLU. TF combinations (1.2:1 and 2.4:1 ratios) stably improved cell viability and reduced LDH leakage across multiple concentrations, reflecting stable synergism between TDS and FLU. In contrast, TE showed unstable synergistic protection, no significant effects at low concentrations, while TM exhibited limited efficacy, with some concentrations even increasing LDH release compared to monotherapy. The stable membrane-protective and cell-viability-enhancing effects of TF explain its superiority, supporting its selection for subsequent *in vivo* studies. These findings are highly consistent with previous studies regarding TDS as an antidepressant enhancer (Antypa et al., 2016; International, 2024), further clarifying the advantages of TF combinations at the cellular level and providing reliable support for subsequent *in vivo* validation.

Pharmacokinetic characteristics are essential indicators for evaluating the rationality of combined medication, and metabolic interactions between drugs directly determine clinical efficacy and safety (Choi and Song, 2026). The present results indicated that both TF-1 and TF-2 markedly improved the oral bioavailability of TDS and FLU, prolonged elimination half-life and mean residence time, suggesting that TF combinations could maintain stable plasma drug concentrations by slowing drug clearance. These results are closely associated with drug metabolism mechanisms: TDS is mainly metabolized by cytochrome P450 (CYP450) isoforms including CYP3A4 and CYP2D6 (Li R. et al., 2023), while FLU not only serves as a substrate of CYP2D6 but also acts as a potent inhibitor of CYP2D6 and CYP3A4 (Maideen et al., 2021). Based on existing literature and our pharmacokinetic observations, the optimized pharmacokinetic profiles of TF combinations may be related to CYP-mediated metabolic interactions via FLU-induced inhibition of CYP450 enzymes, which could partly explain the synergistic effects of TDS and FLU (Yoshino et al., 2002). This discovery enriches the pharmacokinetic research on anxiolytic–antidepressant combinations and provides experimental evidence for clinical regimen optimization and risk reduction of adverse drug interactions.

The chronic unpredictable mild stress (CUMS) combined with single-isolation rearing paradigm was adopted to establish the CAD model *in vivo*. This classic model stably reproduces anxiety- and depressive-like phenotypes by simulating long-term stress exposure in CAD patients, and its pathophysiological characteristics (e.g., hippocampal neuronal damage and 5-HTergic system hypofunction) are highly consistent with clinical manifestations. Hence, it is widely recognized as the gold standard for evaluating anxiolytic and antidepressant agents (Jindal et al., 2013; Wang J.-Y. et al., 2021). In this study, significant reductions in spontaneous activity and aggravated anxiety- and depressive-like behaviors were observed in CAD model mice, which was in line with previous reports (Song et al., 2024; Chen et al., 2025a) and confirmed the successful establishment of the animal model. Furthermore, long-term administration for 28 and 49 days demonstrated that all drug interventions alleviated emotional abnormalities in varying degrees, whereas TF-1 and TF-2 exhibited significantly superior therapeutic effects compared with TDS and FLU monotherapy, with enhanced synergistic benefits following prolonged treatment. These results notially echo clinical findings regarding the synergistic efficacy of TDS combined with SSRIs (Butkevich et al., 2019), but also demonstrate the long-acting superiority of TF combinations through long-duration administration, providing critical experimental evidence for long-term maintenance therapy of CAD.

To elucidate the synergistic mechanism of TF combinations, the core CAD-related signaling pathways, including the 5-HT/5-HT_1_AR and BDNF/TrKB axes, were systematically investigated (Li, 2020; Casarotto et al., 2021). Accumulated studies have illustrated that excessive activation of 5-HT_1_A autoreceptors inhibits 5-HT release via negative feedback, and SSRIs combined with tandospirone can reverse 5-HTergic system dysfunction by targeting this receptor (Kulikov et al., 2018; Zhang R. et al., 2022; Chen R. et al., 2023). In the current study, 5-HT contents and 5-HT_1_AR expression were significantly downregulated in CAD model mice, and TF combination treatment synergistically restored these indicators to normalize 5-HTergic function (Yoshino et al., 2002; Xia et al., 2023). Meanwhile, BDNF/TrKB pathway dysfunction is tightly linked to hippocampal neuronal injury, and both fluoxetine and tandospirone exert regulatory effects on this signaling cascade (Chen Z. et al., 2023; Liu et al., 2023). Our results showed that TF combinations significantly upregulated BDNF expression and TrKB transcription and translation levels. These changes may be associated with improved synaptic plasticity and neuroprotective effects, which are consistent with *in vitro* cellular data and suggest a potential mechanistic link between TF treatment and modulation of these pathways.

Safety is a prerequisite for clinical drug transformation. Considering the long-term medication demand of CAD patients, the impacts of combined regimens on hepatic function, renal function and the central nervous system have attracted extensive attention (M et al., 2025). Previous studies on TDS-antidepressant combinations mainly focused on short-term efficacy and adverse reactions, while long-term organ safety and injury repair capacity remain insufficiently explored (Dong X. et al., 2022). In this study, abnormal hepatic function indicators (ALT and AST) were detected in CAD model mice (Ward et al., 2021). Neither monotherapy (TDS, FLU) nor TF combination treatment induced obvious hepatorenal toxicity. Moreover, TF combinations effectively reversed CAD-triggered hepatic damage. Histopathological examination demonstrated no obvious pathological damage in major organs including the liver, kidney and hippocampus, and abnormal hippocampal neuronal morphology was notably improved in TF-treated groups. These findings fill the research gap in the long-term safety of TDS-FLU co-administration. Combined with its prominent synergistic efficacy, TF combinations were confirmed to possess dual advantages of high efficacy and favorable safety, laying a solid experimental foundation for its future clinical translation and application.

Several limitations of the present study should be acknowledged. First, the present study lacked a single-housing-only control group to separate the independent effects of social isolation from chronic unpredictable mild stress (CUMS); thus, potential behavioral alterations induced by isolation housing cannot be fully excluded, which represents a limitation of this work. Second, PC12 cells are derived from adrenal pheochromocytoma. Although widely used for preliminary neuroprotective screening, they cannot fully represent primary hippocampal neurons or *in-vivo* neural circuits. Future studies using primary neurons, hippocampal neurons or brain organoid models are needed to further validate our findings. Third, In terms of safety evaluation, only serum ALT, AST, CREA, UREA levels and hepatic HE staining were detected with a limited sample size of n = 3 per group. Key toxicity-related indicators including hematological parameters, body weight variation, food intake, and drug accumulation were not assessed in the present study. Therefore, we cannot draw over-optimistic conclusions regarding safety profile, and further comprehensive toxicological assessments are required in future investigations.

## 5 Conclusion

In the present study, two novel compound formulations, TF-1 (TDS: FLU = 1.2:1) and TF-2 (TDS: FLU = 2.4:1), were successfully screened and identified. Both combinations exert multiple superiorities in the treatment of comorbid anxiety and depression (CAD). *In vitro*, they synergistically protect neuronal cells against glutamate-induced injury. Pharmacokinetic results demonstrated markedly enhanced oral bioavailability and prolonged retention time *in vivo*. Animal behavioral tests verified that TF-1 and TF-2 significantly ameliorated anxiety- and depressive-like behaviors in mice, with therapeutic effects further strengthened upon prolonged administration. Mechanistic studies revealed that TF-1 and TF-2 could elevate the contents of 5-HT and BDNF in plasma and hippocampus, upregulate the mRNA and protein expression of hippocampal 5-HT_1_AR and TrKB, and thereby enhance neuronal plasticity and neuronal repair capacity.

In conclusion, TF-1 and TF-2 possess prominent advantages including synergistic therapeutic efficacy, optimized pharmacokinetic profiles, long-lasting effects and favorable safety. This study provides solid experimental evidence and safety support for the research and development of novel compound preparations for CAD intervention.

## Data Availability

All raw data from behavioral tests and western blot are fully presented in the manuscript and supplementary files. The original experimental files are securely stored in the laboratory archive of Southwest Medical University. Researchers with legitimate academic demands may contact the corresponding author to apply for access to the complete raw dataset.

## References

[B1] Antypa N. Vogelzangs N. Meesters Y. Schoevers R. Penninx B. W. J. H. (2016). Chronotype Associations With Depression And Anxiety Disorders In A Large Cohort Study. Depress Anxiety 33, 75–83. 10.1002/da.22422 26367018

[B2] Bandoli G. Campbell-Sills L. Kessler R. C. Heeringa S. G. Nock M. K. Rosellini A. J. (2017). Childhood adversity, adult stress, and the risk of major depression or generalized anxiety disorder in US soldiers: a test of the stress sensitization hypothesis. Psychol. Med. 47, 2379–2392. 10.1017/S0033291717001064 28443533 PMC5595661

[B3] Belus J. M. Muanido A. Cumbe V. F. J. Manaca M. N. Wagenaar B. H. (2022). Psychometric validation of a combined assessment for anxiety and depression in primary care in Mozambique (CAD-MZ). Assessment 29, 1890–1900. 10.1177/10731911211032285 34353141 PMC8816971

[B4] Bobo W. V. Grossardt B. R. Virani S. St Sauver J. L. Boyd C. M. Rocca W. A. (2022). Association of depression and anxiety with the accumulation of chronic conditions. JAMA Netw. Open 5, e229817. 10.1001/jamanetworkopen.2022.9817 35499825 PMC9062691

[B5] Butkevich I. P. Mikhailenko V. A. Vershinina E. A. Barr G. A. (2019). Differences between the prenatal effects of fluoxetine or buspirone alone or in combination on pain and affective behaviors in prenatally stressed Male and female rats. Front. Behav. Neurosci. 13, 125. 10.3389/fnbeh.2019.00125 31244623 PMC6579839

[B6] Camargos Q. M. Silva B. C. Silva D. G. Toscano E. Oliveira B. Bellozi P. (2020). Minocycline treatment prevents depression and anxiety-like behaviors and promotes neuroprotection after experimental ischemic stroke. Brain Res. Bull. 155, 1–10. 10.1016/j.brainresbull.2019.11.009 31756420

[B7] Casarotto P. C. Girych M. Fred S. M. Kovaleva V. Moliner R. Enkavi G. (2021). Antidepressant drugs act by directly binding to TRKB neurotrophin receptors. Cell 184, 1299–1313.e19. 10.1016/j.cell.2021.01.034 33606976 PMC7938888

[B8] Chen R. Lin Q. Wu J. Lin Y. Lin T. Wu W. (2023a). Augmentation therapy with tandospirone citrate in vascular depression patients with mild cognitive impairment: a prospective randomized clinical trial. J. Psychiatr. Res. 159, 274–282. 10.1016/j.jpsychires.2022.12.023 36774768

[B9] Chen Z. Gu J. Lin S. Xu Z. Xu H. Zhao J. (2023b). Saffron essential oil ameliorates CUMS-Induced depression-like behavior in mice via the MAPK-CREB1-BDNF signaling pathway. J. Ethnopharmacol. 300, 115719. 10.1016/j.jep.2022.115719 36126781

[B10] Chen H. Lin Y. Zhao Z. Lin T. Lin Q. Chen X. (2024). Efficacy and safety of venlafaxine hydrochloride combined with tandospirone citrate for patients with vascular depression accompanied by somatic symptoms: an open-labeled randomized control trial. CNS Neurosci. Ther. 30, e14650. 10.1111/cns.14650 38514905 PMC10957720

[B11] Chen J. Wei Y. Li N. Pi C. Zhao W. Zhong Y. (2025a). Preliminary investigation into the antidepressant effects of a novel curcumin analogue (CACN136) *in vitro* and *in vivo* . Mol. Neurobiol. 62, 2124–2147. 10.1007/s12035-024-04363-6 39080204

[B12] Chen J. Yang Y. Jiang F. (2025b). Case report: generalized anxiety disorder and hypertension: a bidirectional loop unraveled by integrated management. Front. Psychiatry 16, 1600910. 10.3389/fpsyt.2025.1600910 41113201 PMC12528110

[B13] Chen M. Li M. Wan D. Zha J. (2026). Global, regional, and national burden of depressive disorders in older adults from 1990 to 2021, with projections of prevalence to 2050: a trend analysis for the global burden of disease study 2021. J. Affect Disord. 405, 121531. 10.1016/j.jad.2026.121531 41780694

[B14] Choi M.-K. Song I.-S. (2026). Pharmacokinetics and drug interactions. Pharmaceutics 18, 67. 10.3390/pharmaceutics18010067 41599173 PMC12845296

[B15] Chrysafi M. Jacovides C. Papadopoulou S. K. Psara E. Vorvolakos T. Antonopoulou M. (2024). The potential effects of the ketogenic diet in the prevention and Co-Treatment of stress, anxiety, depression, schizophrenia, and bipolar disorder: from the basic research to the clinical practice. Nutrients 16, 1546. 10.3390/nu16111546 38892480 PMC11174630

[B16] Crawford C. M. Fallucco E. Fava M. Ingelfinger J. Scott-Vernaglia S. (2024). Depression - screening and treating depression in adolescents. N. Engl. J. Med. 390, e56. 10.1056/NEJMp2400711 38865659

[B17] Demyttenaere K. Kiekens G. Bruffaerts R. Mortier P. Gorwood P. Martin L. (2021). Outcome in depression (II): beyond the Hamilton depression rating scale. CNS Spectr. 26, 378–382. 10.1017/S109285292000142X 32423491

[B18] Dong C. Shi H. Yan Z. Si G. Liu J. (2022a). Quality of evidence supporting the role of non-steroidal anti-inflammatory drugs for the treatment of anxious depression: a protocol for an overview of systematic reviews and meta-analyses. BMJ Open 12, e067621. 10.1136/bmjopen-2022-067621 PMC9806065 36581435

[B19] Dong X. Fang S. Li W. Li X. Zhang S. (2022b). Deanxit and tandospirone relieved unexplained limb edema in a depressed pituitary adenoma survivor: a case report. Front. Psychiatry 13, 965495. 10.3389/fpsyt.2022.965495 36440410 PMC9685525

[B20] Dos Santos M. Lange M. Gervais R. Clarisse B. Capel A. Barillet M. (2019). Impact of anxio-depressive symptoms and cognitive function on oral anticancer therapies adherence. Support Care Cancer 27, 3573–3581. 10.1007/s00520-019-4644-4 30690685

[B21] Duo L.-L. Rao G.-F. (2024). Wuling capsule combined with sertraline in the therapy of anxiety and depression with insomnia in adolescents. World J. Psychiatry 14, 1860–1867. 10.5498/wjp.v14.i12.1860 39704352 PMC11622017

[B22] Fairweather S. J. Fraser H. Lam N. Gilbody S. Paton L. W. Jones H. J. (2026). Prediction models for longitudinal trajectories of depression and anxiety: a systematic review. J. Affect Disord. 401, 121255. 10.1016/j.jad.2026.121255 41621444 PMC7619494

[B23] Ferrer A. Pelegrí A. Labad J. Sagués T. Salvat-Pujol N. de Arriba-Arnau A. (2026). Clock gene influences on sleep quality and HPA axis in major depressive disorder. Sleep. Med. 139, 108730. 10.1016/j.sleep.2025.108730 41429085

[B24] Frey M. I. Aktar E. de Jong P. J. (2026). The presence of and search for meaning in life in comorbid depression and anxiety: a cross-sectional analysis from the Netherlands study of depression and anxiety. J. Affect Disord. 401, 121233. 10.1016/j.jad.2026.121233 41592615

[B25] Fu Y. Ji J. L. Shi S. X. Zhang H. Y. Lin G. Z. Zhang Y. L. (2023). Early outcomes, associated factors and predictive values of clinical outcomes of tandospirone in generalized anxiety disorder: a post-hoc analysis of a randomized, controlled, multicenter clinical trial. Curr. Med. Res. Opin. 39, 597–603. 10.1080/03007995.2023.2175998 36842964

[B26] Gheysens T. Van Den Eede F. De Picker L. (2024). The risk of antidepressant-induced hyponatremia: a meta-analysis of antidepressant classes and compounds. Eur. Psychiatry 67, e20. 10.1192/j.eurpsy.2024.11 38403888 PMC10966618

[B27] Goodwin G. M. (2021). Revisiting treatment options for depressed patients with generalised anxiety disorder. Adv. Ther. 38, 61–68. 10.1007/s12325-021-01861-0 34417993 PMC8437852

[B28] Groen R. N. Ryan O. Wigman J. T. W. Riese H. Penninx B. W. J. H. Giltay E. J. (2020). Comorbidity between depression and anxiety: assessing the role of bridge mental states in dynamic psychological networks. BMC Med. 18, 308. 10.1186/s12916-020-01738-z 32988400 PMC7523307

[B29] Gurbanov A. Kandemir H. Gurbanova L. Gün E. Botan E. Balaban B. (2023). Antidepressant poisoning trends in pediatric intensive care: a comparative study of New- and old-generation antidepressants. J. Clin. Psychopharmacol. 43, 139–144. 10.1097/JCP.0000000000001668 36795032

[B30] Hampf C. Scherf-Clavel M. Weiß C. Klüpfel C. Stonawski S. Hommers L. (2023). Effects of anxious depression on antidepressant treatment response. Int. J. Mol. Sci. 24, 17128. 10.3390/ijms242417128 38138957 PMC10742776

[B31] Huang X. Yang J. Yang S. Cao S. Qin D. Zhou Y. (2017). Role of tandospirone, a 5-HT1A receptor partial agonist, in the treatment of central nervous system disorders and the underlying mechanisms. Oncotarget 8, 102705–102720. 10.18632/oncotarget.22170 29254282 PMC5731992

[B32] Huang S.-S. Chen H.-H. Wang J. Chen W. J. Chen H.-C. Kuo P.-H. (2020). Investigation of early and lifetime clinical features and comorbidities for the risk of developing treatment-resistant depression in a 13-year nationwide cohort study. BMC Psychiatry 20, 541. 10.1186/s12888-020-02935-z 33203427 PMC7672820

[B33] ICD-10 Version (2019). Available online at: https://icd.who.int/browse10/2019/en#/F41.2 (Accessed May 18, 2026).

[B34] ICD-11 for Mortality. (2026). ICD-11 for Mortality and Morbidity Statistics. Available online at: https://icd.who.int/browse/2025-01/mms/en (Accessed March 9, 2026).

[B35] International E. M. (2024). Retracted: clinical analysis on the effects of tandospirone citrate assisted by drawing therapy on medication compliance and sleep quality in patients with anxiety disorders. Emerg. Med. Int. 2024, 9871367. 10.1155/2024/9871367 38298990 PMC10830219

[B36] Jindal A. Mahesh R. Bhatt S. (2013). Etazolate, a phosphodiesterase 4 inhibitor reverses chronic unpredictable mild stress-induced depression-like behavior and brain oxidative damage. Pharmacol. Biochem. Behav. 105, 63–70. 10.1016/j.pbb.2013.01.020 23384434

[B37] Kato M. Hori H. Inoue T. Iga J. Iwata M. Inagaki T. (2021). Discontinuation of antidepressants after remission with antidepressant medication in major depressive disorder: a systematic review and meta-analysis. Mol. Psychiatry 26, 118–133. 10.1038/s41380-020-0843-0 32704061 PMC7815511

[B38] Kazemi R. Rostami R. Rezaei M. Hedayati S. Khomami S. Hadipour A. L. (2025). Comorbid anxiety in depression and rTMS treatment response: a retrospective study. J. Affect Disord. 376, 36–46. 10.1016/j.jad.2025.01.148 39889930

[B39] Kendrick T. (2021). Strategies to reduce use of antidepressants. Br. J. Clin. Pharmacol. 87, 23–33. 10.1111/bcp.14475 32656861

[B40] Kilic B. Bardakkaya M. Ilıkcı Sagkan R. Aksakal F. Shakila S. Dogruer D. S. (2023). New thiourea and benzamide derivatives of 2-aminothiazole as multi-target agents against Alzheimer’s disease: design, synthesis, and biological evaluation. Bioorg Chem. 131, 106322. 10.1016/j.bioorg.2022.106322 36565675

[B41] Knowles K. A. Olatunji B. O. (2020). Specificity of trait anxiety in anxiety and depression: meta-Analysis of the state-trait anxiety inventory. Clin. Psychol. Rev. 82, 101928. 10.1016/j.cpr.2020.101928 33091745 PMC7680410

[B42] Kulikov A. V. Gainetdinov R. R. Ponimaskin E. Kalueff A. V. Naumenko V. S. Popova N. K. (2018). Interplay between the key proteins of serotonin system in SSRI antidepressants efficacy. Expert Opin. Ther. Targets 22, 319–330. 10.1080/14728222.2018.1452912 29542343

[B43] Li Y.-F. (2020). A hypothesis of monoamine (5-HT) - glutamate/gaba long neural circuit: aiming for fast-onset antidepressant discovery. Pharmacol. Ther. 208, 107494. 10.1016/j.pharmthera.2020.107494 31991195

[B44] Li R. Chen Y. Jia M. Jiang X. Wang L. (2023a). Pharmacokinetics and absorption mechanism of tandospirone citrate. Front. Pharmacol. 14, 1283103. 10.3389/fphar.2023.1283103 38027008 PMC10657815

[B45] Li S. Zhu Z. Lan T. Wu Y. Li Y. Wang C. (2023b). Levomilnacipran ameliorates lipopolysaccharide-induced depression-like behaviors and suppressed the TLR4/Ras signaling pathway. Int. Immunopharmacol. 122, 110595. 10.1016/j.intimp.2023.110595 37413934

[B46] Li Y. Du X. An J. Wu H. (2025). Therapeutic drug monitoring for individualized antidepressant treatment. Drug Des. Devel Ther. 19, 11585–11608. 10.2147/DDDT.S566716 41466877 PMC12744603

[B47] Lin J. Su Y. Wang C. Yang F. Xu Y. Yuan Y. (2018). Effects of tandospirone augmentation in major depressive disorder patients with high anxiety: a multicenter, randomized, parallel-controlled, open-label study. J. Psychiatr. Res. 99, 104–110. 10.1016/j.jpsychires.2018.01.020 29433063

[B48] Liu L. Yang W. Lu Y. Wang J. Zheng Y. Gu S. (2023). Clinical efficacy of tandospirone on functional dyspepsia patients with anxiety: a randomized, placebo-controlled study. Dig. Dis. Sci. 68, 521–528. 10.1007/s10620-022-07717-z 36383269

[B49] M D. Cm C. Am A. (2025). Safety in treatment: classical pharmacotherapeutics and new avenues for addressing maternal depression and anxiety during pregnancy. Pharmacol. Reviews 77, 100046. 10.1016/j.pharmr.2025.100046 PMC13095442 40056793

[B50] Maes M. Almulla A. F. Stoyanov D. Zhang Y. (2025). Advancements in the molecular understanding of major depressive disorder uncovering novel targets with therapeutic promise: focus on recurrence of illness. Expert Opin. Ther. Targets 29, 775–791. 10.1080/14728222.2025.2608029 41420146

[B51] Maideen N. M. P. Rajkapoor B. Muthusamy S. Ramanathan S. Thangadurai S. A. Sughir A. A. (2021). A review on pharmacokinetic and pharmacodynamic drug interactions of adrenergic β-blockers with clinically relevant Drugs-An overview. Curr. Drug Metab. 22, 672–682. 10.2174/1389200222666210614112529 34182907

[B52] Meng Y. Li Z. Hou L. Ji Y. (2025). The underlying mechanisms of comorbid anxiety and depression among young women: evidence from brain structure and hormone. Depress Anxiety 2025, 9917994. 10.1155/da/9917994 40959147 PMC12436020

[B53] Monroe S. M. Harkness K. L. (2022). Major depression and its recurrences: life course matters. Annu. Rev. Clin. Psychol. 18, 329–357. 10.1146/annurev-clinpsy-072220-021440 35216520

[B54] Moreno-Fernández R. D. Sampedro-Piquero P. Gómez-Salas F. J. Nieto-Quero A. Estivill-Torrús G. Rodríguez de Fonseca F. (2023). Social avoidance and altered hypothalamic-pituitary-adrenal axis in a mouse model of anxious depression: the role of LPA1 receptor. Behav. Brain Res. 455, 114681. 10.1016/j.bbr.2023.114681 37741054

[B55] Peleg L. C. Rabinovitch D. Lavie Y. Rabbie D. M. Horowitz I. Fruchter E. (2022). Post-SSRI sexual dysfunction (PSSD): biological plausibility, symptoms, diagnosis, and presumed risk factors. Sex. Med. Rev. 10, 91–98. 10.1016/j.sxmr.2021.07.001 34627736

[B56] USP (2010). 2025-2030 Resolutions. Available online at: https://www.usp.org/about/convention-membership/resolutions (Accessed March 9, 2026).

[B57] Rozov A. Fedulina A. Krut’ V. Sokolov R. Sulimova A. Jappy D. (2024). Influence of early-life stress on hippocampal synaptic and network properties. Front. Neural Circuits 18, 1509254. 10.3389/fncir.2024.1509254 39749113 PMC11693662

[B58] Song M. K. Lee J. H. Kim Y.-J. (2021). Effect of chronic handling and social isolation on emotion and cognition in adolescent rats. Physiol. Behav. 237, 113440. 10.1016/j.physbeh.2021.113440 33940083

[B59] Song N. Liu Z. Gao Y. Lu S. Yang S. Yuan C. (2024). NAc-DBS corrects depression-like behaviors in CUMS mouse model *via* disinhibition of DA neurons in the VTA. Mol. Psychiatry 29, 1550–1566. 10.1038/s41380-024-02476-x 38361128

[B60] Yoshino T Nisijima K Katoh S Yui K Nakamura M. (2002). Tandospirone potentiates the fluoxetine-induced increases in extracellular dopamine *via* 5-HT(1A) receptors in the rat medial frontal cortex. Neurochem. International 40, 355–360. 10.1016/s0197-0186(01)00079-1 11792466

[B61] Takeshima M. Enomoto M. Ogasawara M. Kudo M. Itoh Y. Yoshizawa K. (2022). Changes in psychotropic polypharmacy and high-potency prescription following policy change: findings from a large scale Japanese claims database. Psychiatry Clin. Neurosci. 76, 475–477. 10.1111/pcn.13432 35655425 PMC9546399

[B62] Thapar A. Eyre O. Patel V. Brent D. (2022). Depression in young people. Lancet 400, 617–631. 10.1016/S0140-6736(22)01012-1 35940184

[B63] Tran F. D. Bichai G. H. Kravetz Z. Meriden Z. (2025). Management of antidepressant-induced sexual dysfunction: a literature review. Cureus 17, e90170. 10.7759/cureus.90170 40955264 PMC12433685

[B64] Tusa B. S. Alati R. Ayano G. Betts K. Dachew B. (2025). Anxiety and depressive disorders in the offspring of mothers with perinatal depressive disorders. Eur. Child. Adolesc. Psychiatry 34, 4117–4129. 10.1007/s00787-025-02803-9 40711570 PMC12743092

[B65] Vázquez G. H. Baldessarini R. J. (2025). Antidepressants in the treatment of bipolar depression: commentary. Int. J. Neuropsychopharmacol. 28, pyaf013. 10.1093/ijnp/pyaf013 39964358 PMC11929952

[B66] Virtanen S. Kuja-Halkola R. Mataix-Cols D. Jayaram-Lindström N. D’Onofrio B. M. Larsson H. (2020). Comorbidity of substance misuse with anxiety-related and depressive disorders: a genetically informative population study of 3 million individuals in Sweden. Psychol. Med. 50, 1706–1715. 10.1017/S0033291719001788 31328718

[B67] Wang N. Tu Q. (2026). Analysis of factors influencing anxiety and depression disorders among hospitalized patients. Front. Psychiatry 17, 1757945. 10.3389/fpsyt.2026.1757945 41836673 PMC12983223

[B68] Wang C.-C. Du L. Shi H.-H. Ding L. Yanagita T. Xue C.-H. (2021a). Dietary EPA-enriched phospholipids alleviate chronic stress and LPS-induced Depression- and anxiety-like behavior by regulating immunity and neuroinflammation. Mol. Nutr. Food Res. 65, e2100009. 10.1002/mnfr.202100009 34219360

[B69] Wang J.-Y. Zhang Y. Chen Y. Wang Y. Li S.-Y. Wang Y.-F. (2021b). Mechanisms underlying antidepressant effect of transcutaneous auricular vagus nerve stimulation on CUMS model rats based on hippocampal α7nAchR/NF-κB signal pathway. J. Neuroinflammation 18, 291. 10.1186/s12974-021-02341-6 34920740 PMC8680337

[B70] Ward L. D. Tu H.-C. Quenneville C. B. Tsour S. Flynn-Carroll A. O. Parker M. M. (2021). GWAS of serum ALT and AST reveals an association of SLC30A10 Thr95Ile with hypermanganesemia symptoms. Nat. Commun. 12, 4571. 10.1038/s41467-021-24563-1 34315874 PMC8316433

[B71] Xia C.-Y. Zhang N.-N. Jiang H. Lou Y.-X. Ren Q. Zhang X.-L. (2023). Gap junction is essential for the antidepressant effects of fluoxetine. J. Pharm. Pharmacol. 75, 686–692. 10.1093/jpp/rgad016 36892979

[B72] Xie Y. Wu Z. Zhou L. Sun L. Xiao L. Wang G. (2022). Swimming exercise modulates gut microbiota in CUMS-induced depressed mice. Neuropsychiatr. Dis. Treat. 18, 749–760. 10.2147/NDT.S355723 35411144 PMC8994653

[B73] Xu H. Xing S. Lei X. Yi J. Liu S. Du Y. (2022). A famous Chinese medicine formula: yinhuo decoction antagonizes the damage of corticosterone to PC12 cells and improves depression by regulating the SIRT1/PGC-1α pathway. Biomed. Res. Int. 2022, 3714857. 10.1155/2022/3714857 35281603 PMC8916861

[B74] Yang C. R. Zhang X. Y. Liu Y. Du J. Y. Liang R. Yu M. (2020). Antidepressant drugs correct the imbalance between proBDNF/p75NTR/Sortilin and mature BDNF/trkB in the brain of mice with chronic stress. Neurotox. Res. 37, 171–182. 10.1007/s12640-019-00101-2 31493120

[B75] Zhai Y. Boitet L. M. Soldner J. Lockman J. D. Du X. (2025). Trends in clinically significant anxiety, depression, suicidal ideation and service utilisation among US medical students, 2018-2023. BMJ Ment. Health 28, e301528. 10.1136/bmjment-2024-301528 PMC13059911 40425197

[B76] Zhan T.-T. Dong Z.-Y. Yi L.-S. Zhang Y. Sun H.-H. Zhang H.-Q. (2022). Tandospirone prevents stress-induced anxiety-like behavior and visceral hypersensitivity by suppressing theta oscillation enhancement via 5-HT1A receptors in the anterior cingulate cortex in rats. Front. Cell Neurosci. 16, 922750. 10.3389/fncel.2022.922750 36072567 PMC9441562

[B77] Zhang M. Wu W. Huang C. Cai T. Zhao N. Liu S. (2022a). Shuxie-1 decoction alleviated CUMS -Induced liver injury *via* IL-6/JAK2/STAT3 signaling. Front. Pharmacol. 13, 848355. 10.3389/fphar.2022.848355 35462928 PMC9019685

[B78] Zhang R. Li D. Mao H. Wei X. Xu M. Zhang S. (2022b). Disruption of 5-hydroxytryptamine 1A receptor and orexin receptor 1 heterodimer formation affects novel G protein-dependent signaling pathways and has antidepressant effects *in vivo* . Transl. Psychiatry 12, 122. 10.1038/s41398-022-01886-1 35338110 PMC8956632

[B79] Zheng X. X. Li Y. C. Yang K. L. He Z. X. Wang Z. L. Wang X. (2021). Icariin reduces Glu-induced excitatory neurotoxicity via antioxidative and antiapoptotic pathways in SH-SY5Y cells. Phytother. Res. 35, 3377–3389. 10.1002/ptr.7057 33891785

[B80] Zhou L. Wu Z. Wang G. Xiao L. Wang H. Sun L. (2020). Long-term maternal separation potentiates depressive-like behaviours and neuroinflammation in adult Male C57/BL6J mice. Pharmacol. Biochem. Behav. 196, 172953. 10.1016/j.pbb.2020.172953 32450088

